# Advances in Infectious Disease Vaccine Adjuvants

**DOI:** 10.3390/vaccines10071120

**Published:** 2022-07-13

**Authors:** Jingyi Fan, Shengbin Jin, Lachlan Gilmartin, Istvan Toth, Waleed M. Hussein, Rachel J. Stephenson

**Affiliations:** 1 School of Chemistry and Molecular Biosciences, The University of Queensland, St. Lucia, QLD 4072, Australia; j.fan1@uq.net.au (J.F.); shengbin.jin@uq.net.au (S.J.); Lachiegil@hotmail.com (L.G.); i.toth@uq.edu.au (I.T.); w.hussein@uq.edu.au (W.M.H.); 2 Institute for Molecular Bioscience, The University of Queensland, St. Lucia, QLD 4072, Australia; 3 School of Pharmacy, The University of Queensland, Woolloongabba, QLD 4102, Australia

**Keywords:** immunological adjuvants, infectious diseases, pre-clinical and clinical trials

## Abstract

Vaccines are one of the most significant medical interventions in the fight against infectious diseases. Since their discovery by Edward Jenner in 1796, vaccines have reduced the worldwide transmission to eradication levels of infectious diseases, including smallpox, diphtheria, hepatitis, malaria, and influenza. However, the complexity of developing safe and effective vaccines remains a barrier for combating many more infectious diseases. Immune stimulants (or adjuvants) are an indispensable factor in vaccine development, especially for inactivated and subunit-based vaccines due to their decreased immunogenicity compared to whole pathogen vaccines. Adjuvants are widely diverse in structure; however, their overall function in vaccine constructs is the same: to enhance and/or prolong an immunological response. The potential for adverse effects as a result of adjuvant use, though, must be acknowledged and carefully managed. Understanding the specific mechanisms of adjuvant efficacy and safety is a key prerequisite for adjuvant use in vaccination. Therefore, rigorous pre-clinical and clinical research into adjuvant development is essential. Overall, the incorporation of adjuvants allows for greater opportunities in advancing vaccine development and the importance of immune stimulants drives the emergence of novel and more effective adjuvants. This article highlights recent advances in vaccine adjuvant development and provides detailed data from pre-clinical and clinical studies specific to infectious diseases. Future perspectives into vaccine adjuvant development are also highlighted.

## 1. Introduction

Infectious (or communicable) diseases are caused by infectious agents passed from one person (or animal) to another, and these play a huge impact on the health of humans worldwide. These infections are caused by bacteria, parasites, viruses, or fungi (or their toxic by-products), where transmission occurs directly, indirectly or by means of a vector (e.g., mosquitoes). According to the World Health Organisation (WHO), the top ten primary causes of death in low-income countries (which account for 36% of the world’s population) are associated with primary infectious diseases, including malaria, tuberculosis, human immunodeficiency virus (HIV) and influenza (e.g., H1N1 and H5N1), all of which have seriously affected global economies over the past decade(s). More recently, COVD-19 (from the SARS-CoV-2 coronavirus family) was first observed in Wuhan City (China) and has now dominated the worldwide headlines after being deemed a pandemic by the WHO on 11 March 2020.

A review by Baker et al. highlighted the impacts of urbanisation on infectious diseases, and the effects of climatic, technological, and demographic change on disease emergence, dynamics and spread [1]. In previous decades, the transmission of pathogens occurred between wild and/or domestic animals and the human population, causing severe and fatal epidemics worldwide (e.g., HIV-1, HIV-2, the 1918 influenza, and Middle East respiratory syndrome coronavirus). Agricultural and animal husbandry expansion has led to the overuse of antibiotics in domestic animals and pesticides, leading to negative effects on the health of the human population [1].

There is an interface between the ageing population with declining immune function and a potentially increased task of containing infectious diseases that increase the probability of pathogen emergence. With this globally changing landscape, the increased evidence for drug and antibiotic resistance has evolved (e.g., antimalarial resistance) [2]. Although antimicrobials/antibiotics have shown increased efficacy for the treatment of several infectious diseases (e.g., septicemia, meningitis, diphtheria), vaccines are seen as the best method for the long-term prevention (and/or treatment) of infectious diseases worldwide, as vaccines play a key role in limiting disease outbreak and disease burden [3].

Vaccines, a result from pioneering research in the late 18th century by Jenner et al. who investigated the development of a vaccine against the cowpox virus [4], are now the most effective public health strategies used to stimulate protective immunity against infectious diseases worldwide, and have been attributed to saving millions of lives each year [5,6,7,8]. Although a range of successful vaccines have been developed based on attenuated or killed microorganisms (or their toxins), effective vaccines are still absent for the treatment and/or prevention of many infectious diseases known today [9].

With the advancement in vaccine development, from traditional vaccines comprised of whole, killed (or live-attenuated) organisms, to the recent discovery of subunit vaccines using small specific parts of the infectious antigen, vaccines show immense commercial potential with minimal side effects [10]. As the majority of vaccine candidates in clinical development are highly purified proteins and peptides, due to their poor immunogenicity alone (primarily related to the removal of danger signals and their small size), immunological adjuvants (immune stimulants) are required to enhance and/or direct immune responses [11]. However, despite the acknowledged need for novel adjuvants, there are still very few adjuvants in licensed human vaccines.

This review summarises adjuvants in pre-clinical and clinical infectious disease research where adjuvants are evaluated for their role in effective vaccine development, focusing on their pharmaceutical and immunological properties. Adjuvants are classified by their sources, mechanisms of action or chemical properties, where Table 1 identifies the types of adjuvants investigated in the pre-clinical and clinical development of infectious disease vaccines [12,13,14].

## 2. Vaccine Adjuvants

Although somewhat ambiguous, adjuvants are classified by their sources, mechanisms of action, and/or physicochemical properties (Table 1). Vaccine development advances in the post-genomic era have enabled the design of highly pure, safe and simple vaccines. Other vaccine development challenges have emerged in parallel, including the inherent lack of immunostimulatory properties of proteins and peptides. Vaccine adjuvants are therefore considered key components in modern vaccinology, providing the necessary help of enhancing and/or shaping the immune response [14]. Despite vaccines based on whole (or killed) bacteria or viruses being inherently immunogenic against pathogens, adjuvants are required components in vaccines with antigens of low immunogenicity (e.g., peptides, small haptens) [14]. In addition, adjuvants have many other favourable features, including the ability to overcome immune senescence in the elderly, prolonging the memory of a vaccine, broadening the antibody repertoire (including antibody response magnitude and functionality), providing means for dose-sparing, and enhancing effective T cell responses [14]. In general, adjuvants commonly perform two immunological functions: (1) immune-stimulants that intrinsically act on the immune system to improve immune responses of antigens; and (2) vaccine delivery carriers (e.g., emulsions, liposomes, virosomes, virus-like particles, and polymeric nanoparticle adjuvants, and lipid-based nanoparticle adjuvants) which accurately deliver and present vaccine antigens for effective uptake by antigen-presenting cells in a controlled manner and speed to induce and/or enhance an antigen-specific immune response [15].

### 2.1. T Helper Interplay in Adjuvant Function

**Figure 1:**
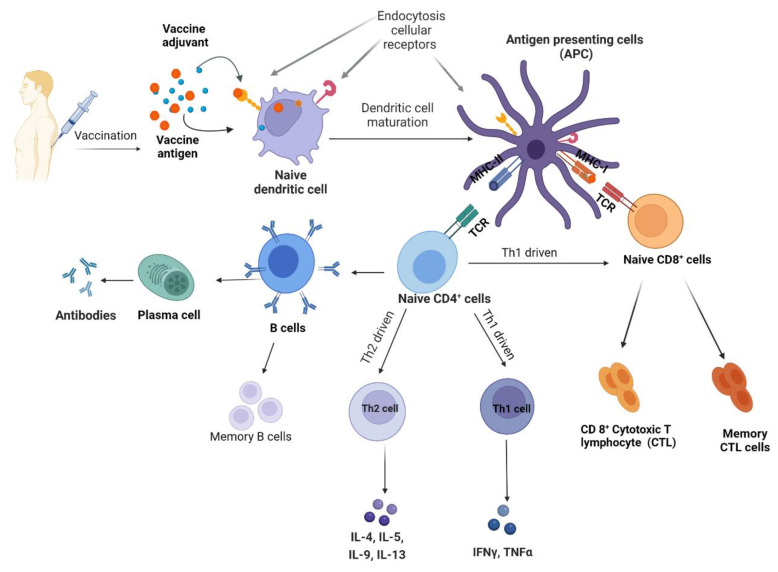
Schematic of an immune response following vaccination. In adaptive immunity, antigens combined with adjuvants are delivered to and bind with naïve dendritic cells, forming antigen-presenting cells (mature dendritic cells), which are recognized by major histocompatibility complex (MHC) class I and MHC-II, thereby binding with T-cell receptors on naïve CD8^+^ cells and naïve CD4^+^ T cells, respectively. Naïve CD4^+^ cells stimulate the production of Th1 (or Th2) responsible for the secretion of different cytokines, and induction of cellular and humoral immunity, respectively.

Adjuvants are essential for enhancing and directing the adaptative immune response to vaccine antigens, mediated by two types of lymphocytes—B and T cells. Upon activation by cytokines, B cells differentiate into memory B cells (long-lived antigen-specific B cells) or plasma cells (effector B cells responsible for the secretion of a large quantity of antibodies). Here, most antigens activate B cells using activated T helper (Th) cells, primarily Th1 and Th2 cells (Figure 1).

Th1 cells secrete interferon (IFN) gamma activating macrophages and induce the production of opsonising antibodies by Th2 cells, leading to a cellular response that protects against intracellular pathogens (invasive bacteria, protozoa and viruses) [16]. The Th1 response activates cytotoxic T lymphocytes (CTL), which induces the death of cells infected with intracellular pathogens. Natural killer (NK) cells are also activated by the Th1 response, and these cells play a major role in the induction of apoptosis in viral-infected cells [17].

Th2 cells secrete cytokines (e.g., interleukins and type I interferon), which induces B cells, leading to the production of neutralizing antibodies [18]. Th2 cells generally induce a humoral (antibody) response, which is instrumental in the bodies defence against extracellular pathogens [19]. Receptors recognizing the Fc-part of immunoglobulins (FcR) play a significant role in responding to infectious diseases and preventing chronic inflammation or auto-immune diseases [20]. Different FcRs recognize and bind different immunoglobulins, transmitting signals into cells. Typical immunoglobulin (Ig) G (IgG), IgM, and IgA are recognized by FcR receptors, controlled by cellular signals evoked from the activation of heterologous receptors in a process generally referred to as inside-out control [21].

As the magnitude and type of Th response to a vaccine is greatly modulated through the use of adjuvants, as our understanding of the mechanisms of ‘immunogenicity’ and ‘adjuvancy’ increases, new adjuvants and adjuvant formulations are being developed and/or optimised.

### 2.2. Importance of Toll-like Receptors in Adjuvant Function

Toll-like receptor (TLR) agonists form a large adjuvant family. They have been shown to play a key role in stimulating both innate and adaptive immunity for infectious disease vaccine development (Table 1) [22]. Toll-like receptors, as a family of pattern recognition receptors, are major sensors of the innate immune system to recognise invasive pathogens [23]. In 1988, TLRs were initially identified as factors involved in the embryonic development and resistance of the fly *Drosophila* to bacterial and fungal infection, and animals as low as nematodes (e.g., *Caenorhabditis elegans* and Ciona intestinalis) have also found TLRs [23,24]. TLRs are derived into 10 TLRs in humans (TLR1–TLR10) and 12 mice TLRs (1–9 and 11–13) [25]. TLRs are on the cell surface (TLR1, 2, 4, 5 and 6) or intracellular compartments (TLR 3, 7, 8 and 9) [25]. TLRs recognise distinct structures (agonists) in microbes, referred to as pathogen-associated molecular patterns or danger-associated molecular patterns [23]. Ligand binding of these structures to TLRs invokes a cascade of intracellular signalling pathways that induce the production of factors involved in inflammation and immunity [23]. These agonists target a broad range of common motifs found in pathogenic bacteria, viruses, parasites and fungi, including nucleic acids, lipopeptides, peptidoglycans and lipoproteins [22]. The primary advantage for TLR agonist adjuvants (expressed on macrophages and dendritic cells) is their ability to activate signalling pathways upon ligand binding, stimulating innate and adaptive immunity [26]. The design of small-molecule TLR agonist adjuvants has immense potential for the eradication of inflammation, cancer, infection, and autoimmunity [27]. For example, a combination of TLR1/2 and TLR3 adjuvants (L-pampo) combined with SARS-CoV-2 antigens, forming the COVID-19 vaccine candidate [28]. Jeong’s group administered this vaccine candidate to female BALB/c mice and female ferrets [28]. It is reported that L-pampo adjuvant stimulates strong humoral and cell-mediated immune responses against SARS-CoV-2antigens [28]. The antibody level was higher than other adjuvants (e.g., alum, AddaS03TM, and alum combined with CpG [28]. L-pampo did not only produce neutralising antibodies but also retained protective effects in ferrets [28].

TLR agonists have immense potential in clinical infectious disease vaccine research. TLR agonists uses are limited by the overexpression of inflammatory responses in certain signalling pathways, leaving a pronounced effect on the strengthening of inflammatory diseases [22]. No specific activation of immune cells as anti-infectious or anti-tumour agent carriers is possible to active self-clones, resulting in autoimmune response [29]. Another drawback of TLR agonists increases the risk of the susceptibility to tumours [29]. Therefore, strict regulation is required for the use of TLR agonists as vaccine adjuvants for infectious diseases, especially for humans, in order to minimise the risk of adverse effects. In recent studies, Flagelline, Pam_3_CSK_4_, L-pambo derivatives as TLR 1/2 agonists, GLA-AF, Monophosporyl lipid A (MPL) as a TLR4 agonist, Resiquimod (R-848) as a TLR7/8, and, oligodeoxynucleotides of cytosine and guanine with phosphodiester backbone (CpG ODNs) as a TLR9 agonist showed improved efficacy and safety in pre-clinical and clinical trials [27].

## 3. Mineral-Based Adjuvants

Aluminium salts (100–1000 nm insoluble gel-like particles) have a long history of use in adjuvant discovery, with most showing excellent safety profiles in human vaccines, starting with alum adjuvants in diphtheria and tetanus vaccines of the 1930s [30,31,32]. Commercial sources of aluminium adjuvants include aluminium phosphate, aluminium hydroxide, amorphous aluminium hydroxy phosphate sulfate, aluminium hydroxyphosphate sulfate, or a mixture of aluminium and magnesium hydroxides, with different physicochemical properties (including size and charge) [33].

Alum has the capacity to stimulate strong humoral responses (Th2), although the interaction of alum with the immune system is not well defined. Alum adjuvants allow for a short-term depot effect, which enables the controlled release of the antigen(s) at the site of injection [34]. This depot formation occurs following electrostatic interactions (between the soluble antigen and alum), which indirectly prolongs antigen release at the injection site, while hydrogen bonding, Van der Waals forces, hydrophobic interactions, and ligand exchange also play important roles [35]. Further, studies demonstrated that an antigen-alum depot diminished 14 days following immunisation without any effect on the immune response [36]. Conversely, opposing claims are also reported on alum’s role in the instigation of this depot effect [36].

Alum salts have the potential to stimulate the Nod-like receptor (NLR) family, pyrin-domain-containing 3 (NLRP3) inflammasome activation [34]. Several studies have concluded that alum adjuvants induce NLR3 inflammasome-induced caspase-1 activation, and secreted IL-1β, IL-18 and IL-33 cytokines to their bioactive forms, which affected alum-mediated cellular recruitment, dendritic cell maturation, antigen uptake, and secretion of T cells [34,36]. However, the role of inflammasomes in adjuvant activity of alum salts is still under debate [36]. The irritable factor of alum adjuvants directly triggers T helper type 2 (Th2) immune responses, improving the immune-stimulatory activity of the alum-adjuvanted vaccine through the recruitment of immunoglobin (Ig) G1, IgE, IL-4, IL-5, and eosinophils.

As an adjuvant, aluminium phosphate has a higher affinity with antigens compared with aluminium hydroxide, simply due to a stronger ligand exchange in vivo [37]. Here, the affinity of alum adjuvants is modified via pre-treatment of alum salts with the appropriate phosphate buffer [37]. Extended research into aluminium salts has enhanced the understanding of their mode of action for use as vaccine adjuvants and has resulted in their extensive use in the development of infectious disease vaccines (Table 2).

Alum salts are inexpensive, and safe and simple to formulate, store and transport, but are generally weaker adjuvants than modern-day, emulsion-type adjuvants [15]. Alum adjuvants, (e.g., TWINRIX^®^, PEDIARIX^®^, Alhydrogel^®^ and Adju-Phos^®^) have regulatory approval from the United States (US) Food and Drug Administration (FDA) for use in 25 commercial infectious disease vaccines (e.g., Hepatitis A, influenza A, and meningococcal Group B), although they are most prominently used in diphtheria and tetanus vaccines where they are combined with toxoid and inactivated organisms (Table 2) [34,35].

Limitations around mineral-based adjuvants include insufficient immunoprecipitation (when compared with other adjuvant classes), a low production of Th1-mediated and cytotoxic T lymphocyte (CTL) cellular responses, and an enhancement of the eosinophil and IgE secretions leading to an increased risk of allergic reactions and/or anaphylaxis [108]. As mineral-based adjuvants are non-biodegradable, patients with impaired renal function have been shown to elicit nervous system disorders and bone diseases due to the systemic accumulation of aluminium minerals [108].

Calcium phosphate (CaP), initially used as an adjuvant in France for diphtheria, tetanus, pertussis and poliomyelitis vaccines, was completely substituted by alum salts in the late 1980s. Today, CaP adjuvants remain an approved WHO adjuvant for human vaccination and are considered a safe replacement for aluminium salts in vaccines. CaP adjuvants have been shown to elicit humoral, cellular and mucosal immune responses against pathogens, with excellent biodegradation properties [45]. Compared with aluminium-based adjuvants, CaP adjuvants enhance the secretion of IgG1 antibodies with the reduction of IgE antibodies [109].

### 3.1. Pre-Clinical and Clinical Development of Mineral-Based Adjuvants

With the success of various alum-based adjutants in commercial vaccines, clinical research into mineral-based adjuvants continues to explore their function, mechanism and safety profile in greater depth (Table 2) [110].

#### 3.1.1. Aluminum Adjuvants

While alum adjuvants have been widely used in vaccine development for decades, the host metabolic pathway for these adjuvants is not completely understood [111]. Pre-clinical research by Khoomrung et al. assessed levels of lipid concentration in the serum of mice following vaccination with a tuberculosis (*M. tuberculosis* H56 fusion protein) vaccine containing 2% alhydrogel (Table 2) [111]. This research identified that lipid metabolism was involved in alum-adjuvanted vaccine uptake after subcutaneous administration into mice [111]. Here, the difference of metabolite and lipid was identified in mice vaccinated with H56 fusion protein plus alum, and H56 fusion protein alone [111]. It was reported that the lipid metabolic response of triglycerides (TAGs) in H56 plus alum-treated mice significantly increased at 24 h compared to 6 h post vaccination [111]. Lipid metabolism of TAGs with long-chain unsaturated fatty acids decreased 24-h post vaccination, and short-chain fatty acids 168 h post vaccination compared to antigen alone [111]. Recent evidence highlighted that both fatty acid synthesis and lipid oxidation significantly affected immune cell differentiation and function [111]. Further studies are required to evaluate whether the lipid oxidation and fatty profile stimulated upon alum adjuvant administration is representative of the characteristic Th2 immune response and how it compares with other adjuvants [111].

The primary limitation of alum adjuvants is their stimulation of IL-10, which inhibits Th1 responses [112]. Mice injected with alhydrogel combined with Leishmania major antigens (a recombinant polyprotein derived from Leishmania species, MML polyprotein) to determine the total number of live cells and IL-10-positive cells in the peritoneal cavity, draining lymph nodes and spleen showed a rapid production of IL-10 following intraperitoneal injection when compared to mice treated with PBS alone [112]. As Th1 immune responses are vital for the elimination of several infectious diseases (e.g., HIV, *Streptococcus pneumoniae*, *Staphylococcus aureus*, *Bordetella pertussis*), alum’s involvement in the generation of IL-10 secreted by stimulated dendritic cells and macrophages, and the potential inhibitory effect of alum on Th1 responses, therefore prevents alum from being a universal vaccine adjuvant [112].

Alum’s immunogenicity and safety continue to be strong advantages for its use in novel infectious disease vaccines [113]. A Phase 1 clinical study of a respiratory syncytial virus (RSV) vaccine containing alum was completed in 2017 (NCT01905215) (Table 2) [39]. In this Phase 1 clinical trial, purified recombinant RSV protein F vaccine, engineered to preferentially maintain prefusion conformation (RSV-PreF) formulated with two alum-adjuvanted formulations (30 μg RSV-PreF/alum and 60 μg RSV-PreF/alum), was immunised into 128 healthy volunteers, analyzing for safety and immunogenicity [39]. Immune responses to this RSV-PreF vaccine were enhanced in the alum-adjuvanted formulations, while immune responses declined in all study groups from day 60 post vaccination, highlighting that the timing of vaccination plays an important role in disease protection [39]. Of the two alum-adjuvanted RSV vaccines, aside from common vaccine side effects (e.g., pain at the injection site, fatigue), there were no significant differences in terms of safety and reactogenicity between the vaccine formulations, and no vaccine-related serious adverse events or withdrawals reported [39].

In addition, a Phase 2 study of a recombinant RSV fusion protein vaccine (RSV F) co-administered with aluminum phosphate was tested for safety and immunogenicity in females of childbearing age (NCT01704365; Table 2) [42]. It was concluded that all vaccines were tolerated and the most common adverse effects (injection-site reactions, moderate pain, fatigue, tolerated headache) were acceptable. Immunisation of RSV F vaccine combined with aluminum phosphate enhanced the stronger immune response with protection antibodies in comparison to vaccine alone [42]. Based on successful outcomes of the Phase 2 clinical trial, a Phase 3 clinical trial of RSV F vaccine was conducted in pregnant females (NCT02247726) and demonstrated that infants born to pregnant women administered an RSV vaccine had higher levels of anti-F immunoglobulin G (IgG) and palivizumab-competing antibody at birth compared with infants of women who received the placebo vaccine [39]. Here, tolerance of this respiratory Syncytial Virus Fusion (F) protein vaccine (RSV F) was better, with only mild and transient injection site pain, similar to that reported for the placebo group [39].

Due to the rapid mutation of HIV on the human immune system, the development of a safe and effective HIV vaccine has proven to be extremely difficult [114]. The discovery of broadly neutralising antibodies in HIV patients prompted research into the investigation of the HIV-1 envelope as a vaccine antigen [115]. The HIV envelope glycoprotein (Env) spikes are a viral-coded antigen exposed on the virus surface, triggering the secretion of neutralising antibodies [115]. HIV vaccine design has focused on the generation of soluble Env trimer mimetics to overcome the inherent instability and flexibility of naïve Env trimers [114]. Over the last few decades, the safety and efficacy of HIV-1 vaccines have been tested in early-phase human trials leading to the selection of several envelope proteins (e.g., 1086.C gp120, 1086.C gp140, BG505 SOSIP.644 gp140) as promising HIV vaccine candidates [116].

In 2005, AIDSVAX B/E (an HIV vaccine candidate containing the gp120 antigen and adjuvanted with alum) failed a Phase 3 clinical trial (NCT00006327; Table 2) as it unsuccessfully prevented HIV infection [43]. In 2013, optimised HIV-derived BG500 SOSIP, gp140 trimers were shown to secrete neutralising antibodies, eliminating the majority of the neutralisation phenotype of circulating HIV viruses (Titer 1), neutralising autologous virus strains (Titer 2), and some heterologous (Titer 2) HIV viruses [116]. In 2019, HIV Env trimers (BG505 SOSIP.664 gp140) co-administered with alum provided an enhanced immunogenicity and safety profile in pre-clinical development when compared to the Env trimers alone, and from this outcome, this vaccine was assessed in Phase I clinical studies (NCT04177355) with an unknown outcome [116,117].

The outbreak of coronavirus disease in the year of 2019 (COVID-19) caused by the SARS-CoV-2 (severe acute respiratory syndrome coronavirus 2) pathogen caused a major impact on the health and economics of humans globally [118]. With vaccination seen as the primary method for preventing the further spread of this deadly infectious disease, the identification of the glycosylated spike protein on the coronavirus viral surface was shown to be essential for viral entry into a host’s cell [119]. Consequently, this spike protein was the primary target for human immune antibodies and worldwide collaborative vaccine development [120].

Inactivated SARS-CoV-2 vaccine (PiCoVacc) adjuvanted with alum salts enhanced S-specific, receptor-binding domain (RBD)-specific, and N-specific IgG in rodents [38]. Further, T cell responses elicited by any SARS-CoV2-2 vaccine would have been well controlled to eradicate immunopathology [38]. Based on this pre-clinical assessment, the PiCoVacc vaccine was assessed in Phase 3 clinical studies (NCT04456595), again inducing RBD-specific IgG and neutralising antibodies, but without any specific cellular immune response in humans [38]. Assessment of the antigen without alum was not reported.

One of the leading COVID-19 subunit vaccine candidates, SCB-2019, was a recombinant SARS-CoV-2 fusion protein engineered by Trimer-Tag technology in a mammalian cell line [44]. This antigen was clinically assessed in Phase 1 clinical trials in conjunction with either AS03 or alum/CpG adjuvants (NCT04405908; Table 2) formulated with different doses of the SCB-2019 antigen [44]. Adjuvanted SCB-2019 (AS03 or CpG/Alum) at the tested dose levels secreted higher IgG titres and stronger immunogenicity compared to non-adjuvanted SCB-2019 [44]. Following two immunisations, both CpG/Alum and AS03-adjuvanted SCB-2019 vaccines showed enhanced Th1-polarised and CD4^+^ T cell responses, with the secretion of protective antibodies against SARS-CoV-2 [44]. Pathogenic antigens adjuvanted with CpG/Alum enhanced a lower dose-dependent immune response than the AS03-adjuvnated vaccine, especially in the elderly vaccine group [44].

Current pre-clinical and clinical research continues to improve our understanding and application of alum as a vaccine adjuvant for infectious diseases. Alum’s continued, prominent use in novel vaccine trials, including recent COVID-19 vaccines, is a testament to its efficacy and safety. Here, alum induces moderate-strong IgG antibody responses in humans but fails to produce a cellular immune response [35,121]. Although advanced studies have increased knowledge of alum as a vaccine adjuvant, research into the induction of a cellular response and biological function (e.g., IL-1β and neutrophil accumulation) is still vital for advancing its role in global vaccine development for infectious diseases [122].

#### 3.1.2. Calcium Phosphate Adjuvants

Calcium phosphate (CaP) adjuvants have been explored in several pre-clinical vaccine trials against infectious diseases (Table 2).

Human foot-and-mouth disease virus (FMDV) is the most common cause of fatality in the Asia-Pacifica region (including Malaysia, Singapore and Japan) [47]. Since its discovery in 1969, millions of children (aged between 5 and 10 years of age) are affected by this infectious disease [47]. Human enterovirus-71 (also referred to as Enterovirus A71 or HEV-71), is a virus of the genus Enterovirus in the Picornaviridae family, notable for its role in causing epidemics of severe neurological disease and hand, foot, and mouth disease in children. Unfortunately, vaccines and effective antiviral therapy is not yet available on the market to prevent this disease [47].

Saeed and co-workers demonstrated that inactivated HEV-71 co-administered with CaP stimulated enhanced levels of EV-7-specific and protective IgG antibodies compared to the antigen alone, following intradermal administration into rabbits [47]. Further, this pre-clinical trial showed that a nano-sized (73 nm) CaP adjuvant produced higher HEV-71 antibody titres with significant protective immune responses compared to micro-sized (1.7 µm) CaP adjuvant [47]. In addition to this, Jopappa and co-workers vaccinated with the FMDV’O’P1-3CD DNA antigen (which includes all structural and non-structural genes from the foot-mouth disease viral capsid) encapsulated in CaP in nanoparticle size (50–100 nm diameter in size) (Table 2) [49]. Following intraperitoneal administration into mice and guinea pigs, the CaP-encapsulated FMDV DNA vaccine induced strong, significant cell-mediated and humoral immune responses when compared with the naked (un-adjuvanted) DNA vaccine [49]. Although lower neutralising antibody titres were observed for this nanoparticle DNA vaccine compared to conventional FMD vaccines (chemically inactivated whole virus antigen combined with either alum or saponin adjuvant), in this pre-clinical trial, higher protection from the FMD virus was observed for mice (100%) and guinea pigs (87.5%) [49]. This vaccine shows efficacy potential, however, to date no clinical trials have been recorded.

Another debilitating infectious disease is herpes simplex virus (HSV) which belongs to the alphaherpesviruses subfamily of herpesviruses, including type-1 (HSV-1) and type-2 (HSV-2) [123]. HSV-1 primarily induces oral and ocular infections, with HSV-2 inducing genital infections. Both HSV-1 and HSV-2 cause severe infections on the nervous system and neurological diseases (e.g., blindness, meningitis, and encephalitis) [123]. One research group working on HIV vaccines, He et al., assessed a CaP-nanoparticle-encapsulated vaccine containing the inactivated HSV-2 viral protein [45]. Following intraperitoneal administration in mice, the CaP-adjuvanted HSV-2 vaccine stimulated an increased IgG2a response with effective protection against live HSV-2 infection and a reduction in IgE responses when compared to an alum-adjuvanted vaccine formulation [45]. To date, only pre-clinical data has been reported for a mineral-based adjuvanted HSV vaccine, with other adjuvant classes (e.g., AS04, Matrix-M, and QS-21) progressing to clinical trials (Table 2).

Despite an unknown mechanism of action for the CaP adjuvant, it is believed to act similarly to an aluminum adjuvant, allowing for the depot effect and accounting for a slow release of antigens over time [45]. With the encapsulation of the pathogenic agent (e.g., HIV-1 viral protein) in CaP nanoparticles, host cells effectively uptake the complex, facilitating subsequent antigen expression [45]. Consequently, CaP adjuvants will play a huge potential in the future design of single-dose vaccines with sustained-release capabilities due to their simple formulation, physicochemical properties, and ability to be used for vaccines requiring different routes of administration [45]. CaP nanoparticles have been widely approved in clinical trials for diagnostic purposes and bone regeneration, with clinical trials to assess CaP nanoparticle vaccines projected to start in the upcoming years [46].

#### 3.1.3. Other Mineral Adjuvants

In addition to aluminum and calcium adjuvants, other metal salts (e.g., iron and beryllium) have been used as vaccine adjuvants. For example, iron oxide (IO) nanoparticles (<20 nm), with their excellent safety profile and low cost of production, were used as a vaccine delivery platform in the hunt for a vaccine against malaria [124]. Pusic and colleagues selected the merozoite surface protein 1 (rMSP1) as a recombinant malaria antigen and adjuvanted with IO nanoparticles (rMSP1-IO), and following subcutaneous administration in a murine model, this pre-clinical trial reported 100% responsiveness with antibody titres (IgG), with the induction of high levels of parasite-inhibitory antibodies compared to mice with rMSP1 adjuvanted with Montanide ISA-51 [124].

## 4. Microbial/Bacterial Adjuvants

### 4.1. Flagellin Adjuvants

Flagellin is the structural component (subunit protein) of flagellum, a whip-like locomotor organ found on Gram-negative bacteria [125]. Flagellin consists of highly conserved N- and C-terminal domains (D1 and D2 domains) with an intervening hypervariable region (D3 domain) [125]. Each filament contains over 20,000 subunits of monomeric flagellin (Flic) [126]. With strong oligomerisation potential, flagellin polymerises into filaments in vitro, stimulating innate and adaptive immune responses via the germ line-encoded pattern recognition receptor, toll-like receptor (TLR) 5. Here, flagellin’s D1 and D2 domains are essential in TLR5 identification, stimulating a pro-inflammatory response [125,126]. Flagellin contributes to the activation of TLR5 on both B and T cells, enhancing a long-term, T-cell-dependent antibody immune response, while polymeric flagellin adjuvants activate B cells by crosslinking B cell receptors to stimulate a humoral IgM immune response without the help of T cells [125].

Flagellin also stimulates the secretion of pro-inflammatory cytokines and chemokines in a large number of innate and non-immune cells (e.g., B and T cells, dendritic, natural killer and epithelial cells,) which leads to activation of an antigen-specific adaptive immune response [125]. A higher concentration of chemokines and cytokines in draining lymph nodes was achieved from the use of flagellin as a vaccine adjuvant, maximising the opportunity for antigen-specific lymphocytes encountering their antigens [125].

#### Pre-Clinical and Clinical Development of Flagellin Adjuvants

The VAX-102 influenza vaccine (Table 2) contains the influenza A viral matrix protein (M2e) antigen, a small, non-glycosylated ectodomain of 24 amino acids, adjuvanted with flagellin isolated from *Salmonella typhimurium* [25]. VAX-102 was shown to stimulate stronger immune responses against influenza virus when compared to the un-adjuvanted antigen following intramuscular immunisation in mice [25]. Phase I clinical trials of VAX-102 (NCT00603811) in healthy volunteers concluded promising safety data in humans with the M2e/flagellin vaccine shown to enhance a strong immune response and maximise post-vaccination protection response [27]. Outcomes from this trial hinted at an improved influenza A vaccine for the elderly population, with the current influenza A vaccine requiring annual immunisations with the induction of new immune responses, which at present, significantly deteriorates with age [27].

Vaccine development for the elderly population is a major challenge to public health services due to an inevitable ageing immune system [127]. This is caused by the malfunction of innate immune system cellular receptors, reduction of naïve T cells, alteration of T cell composition population, and replicative senescence of memory cells [127]. As a result, the efficacy of vaccination in the elderly is significantly decreased when compared to the younger population. It is therefore necessary to design disease-specific vaccines for the elderly, keeping in mind the importance of induction of long-term and protective immune responses [127].

VAX-125 combines the globular head (amino acid 62-84) of the hemagglutinin (HA) 1 domain of the influenza A hemagglutinin (a major influenza vaccine antigen) with flagellin, and this vaccine was designed to protect the elderly population against seasonal influenza [52]. VAX-125 was assessed in Phase I clinical trials in healthy people aged over 65 years of age (NCT00966238) and in young adults aged between 18 and 49 years of age (NCT00730457) [51,52]. Outcomes for both trials indicated that intramuscular administration of VAX-125 was well tolerated at all dose levels, with mild to moderate pain at the injection site and no serious adverse events. It was demonstrated that young healthy adults secreted IgG antibodies and induced a functional humoral immunity against native virions [121]. Furthermore, VAX-125 produced an enhanced (10-fold) increase in IgG antibody levels that remained as post-vaccine protection in the over-65-years-old vaccine group [120]. Flagellin is therefore seen as a useful and effective adjuvant to eradicate immunosenescence in the elderly population, leading to VAX-125 being a promising new vaccine candidate for prevention of the influenza A epidemic in both the young and the elderly populations. Further, the VAX2012Q vaccine (Table 2) contained four seasonal influenza strains (HA1-2 subunit from H1N1 influenza A; 57-amino acid protein of the HA1 globular head from H3N2 influenza A; HA1 subunit from Yamagata influenza B; HA1-2 subunit from Victoria influenza B) each linked with a flagellin adjuvant. This vaccine completed Phase I clinical studies as a seasonal influenza vaccine in adults, and is now in Phase II clinical studies (NCT02434276; Table 2) [25]. Outcomes for the Phase 1 studies (NCT02015494; 300 healthy adults; aged between 18 and 40) tolerated the VAX2012Q vaccine at all dose levels with no adverse events outside of mild injection-site pain and transient chills and fevers. VAX2012Q produced IgG antibodies and enhanced the humoral immune response at all dose levels, with a protective immune response [53].

Plague, an acute and fatal infectious disease of both humans and animals, is induced by the Gram-negative coccobacillus, *Yersinia pestis*, where to date, no commercial vaccine protects against pneumonic plague [54]. Mizel and co-workers designed a recombinant protein of two protective antigens (F1 and V protein) of *Y. pestis*, which was then combined with the hypervariable region of flagellin, forming the final vaccine candidate (flagellin-F1-V). Following intranasal immunisation into mice and two species of non-human primates, potent anti-F1 and anti-V humoral immune responses were reported in all animal species with the bacteria completely cleared within 3 days post challenge, indicating significant post-vaccination protective immune response compared to the un-adjuvanted vaccines [54,128]. Following these outstanding results, a Phase I clinical trial (NCT01381744) of the flagellin-F1-V recombinant fusion protein intramuscularly immunised into 45-year-old healthy volunteers was commenced; however, to date, no further information is available from this trial [54].

### 4.2. Lipopolysaccharide Adjuvants

**Figure 2:**
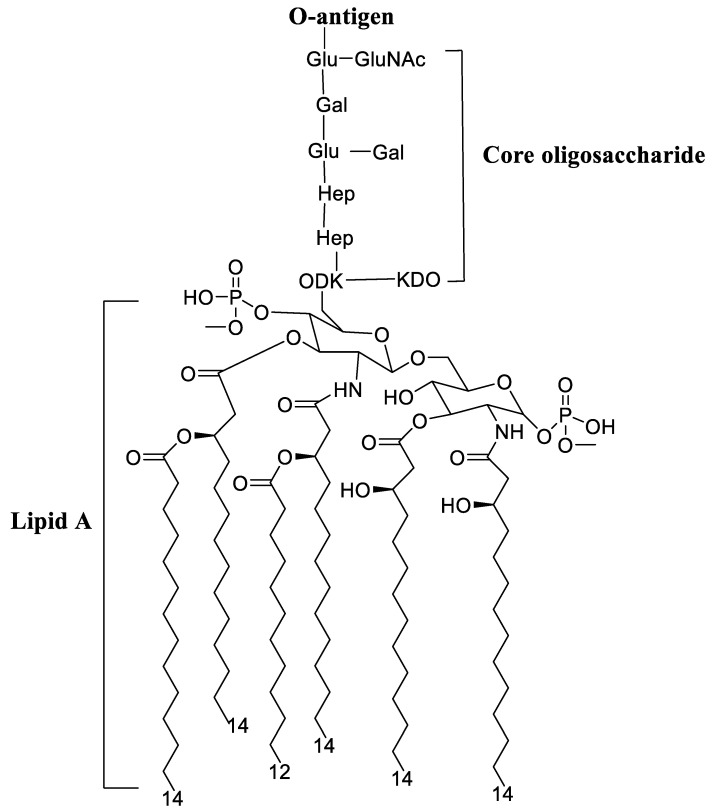
Structure of *E. coli* lipopolysaccharide. Abbreviations Gal: D-galactose; Glu: D-glucose; Hep: L-glycero-D-manno-heptose; KDO: 3-deoxy-D-manno-oct-2-ulosonic acid; P: phosphate.

Lipopolysaccharides, endotoxins on the outer membrane of Gram-negative bacteria, are comprised of three parts: a polysaccharide O-antigen, a core-oligosaccharide and a hydrophobic lipid A (Figure 2) [129,130].

The endotoxin derived from many Gram-negative organism lipopolysaccharides (including bacterium and viruses) binds pattern recognition receptor complexes (e.g., TLR4, CD14) and myeloid differentiation protein 2 (MD-2; a 25-kD glycoprotein), leading to the activation of immune cells and production of inflammatory cytokines, which in turn induces an innate and adaptive immune response against the pathogenic antigen [130,131]. As an adjuvant, lipopolysaccharides have been shown to induce a Th1-biased immune response, however, modification of the lipopolysaccharide structure triggers an immune response required in vaccines against specific pathogens while at the same time lowering their toxicity [130].

Chemical treatment of lipopolysaccharide in conjunction with genetic modification of the lipopolysaccharide’s biosynthesis pathway has led to a promising reduction of endotoxin present, whilst still maintaining the lipopolysaccharides immune-stimulatory properties. Examples of modified lipopolysaccharide derivatives important for adjuvant development include MPLA (monophosphoryl-lipid A) and GLA [130].

#### Pre-Clinical and Clinical Development of Lipopolysaccharide Adjuvants

Due to the strong immunogenic potential of TLR agonists, novel routes of administration have been at the forefront of vaccine research [25]. TLR4, the first toll-like receptor identified in mammals, plays a pivotal role in the inflammatory pathway, making it an ideal agonist for adjuvant development [25].

MPLA is the biologically active part of Gram-negative bacterial lipopolysaccharide endotoxin and a TLR4 agonist and has been approved for use as a human adjuvant in several FDA and European vaccines [132]. For example, MPLA has been licensed in the hepatitis B vaccine, Fendix^®^ (pathogenic DNA sequences selected from hepatitis B virus), which is widely used in renal failure patients, and the human papilloma virus (HPV) vaccine, Cervarix^®^ (virus-like particles from HPV-16 and HPV-18), which is highly efficacious in the reduction of persistent infections and cervical lesions (Table 2) [133]. Interestingly, the clinical-grade form of MPLA (3-O-desacyl-4′-monophosphoryl lipid A [MPL] adjuvant™) is incorporated with other adjuvants (e.g., alum, adjuvant system [AS] 01, AS02) forming a combination adjuvant to improve the “mechanical” delivery of the vaccine antigen (Table 2) [134].

GLA, a synthetic TLR4 agonist, is purified from *Salmonella minnesota* as a detoxified bacterial lipopolysaccharide and is a hexa-acylated molecule [135]. Compared with heterogeneous MPLA, as an adjuvant, GLA is more potent on a molar basis when tested on the activation of human dendritic cells and peripheral blood mononuclear cells, enhancing the immunogenicity of co-administered recombinant antigens, allowing for cytokine secretion (e.g., IL-1, IL-10, and INF-α) and producing cell-mediated and Th1 immune responses [135,136].

The skin is an effective administration site for vaccine administration and can be used as an alternative to traditional immunization routes such as subcutaneous, intramuscular or intradermal delivery [137]. Intradermal vaccine technology (e.g., microneedle patches that contain a layer of dried antigen on their surface that becomes embedded within the skin through the microneedles following vaccination) is capable of producing equivalent antibody responses at lower doses in strength compared with other administration routes, and this is referred to as “dose-sparing” [138]. These microneedle patches are easy to self-administer onto the skin, relieving the necessity for trained health professionals to deliver the vaccine [55]. Rabies, a zoonosis, has occurred in over 100 nations worldwide and is a fatal disease once symptomatic. However, the cost of a full dose of the rabies vaccine by intramuscular administration limits its widespread application in low-income countries [138]. Intradermal administration of the rabies vaccine offers an equivalently safe and immunogenic vaccine that only requires 20% of the dose for post-exposure prophylaxis, significantly reducing the cost of the traditional vaccine by 60% to 80% [138]. Consequently, intradermal vaccination of rabies has been accepted and used in India, Sri Lanka and Thailand, with approximately 15 million doses administrated annually. [138,139]. Interestingly, immunisation of the same Hepatitis B vaccine using different routes of administration (intramuscular and intradermal) to patients showed that intradermal administration presented a higher immunogenicity than intramuscular administration, with significantly better seroconversion rates than that of the intramuscular-administered vaccine [138].

However, several microneedle types (e.g., coated or dissolved) have been utilized in vaccine delivery, releasing the dry antigen into the epidermis and dermis, without controlling antigen release [137]. Du and co-workers developed a hollow microneedle, which was used to deliver a model antigen (ovalbumin) combined with four different adjuvants (including PLGA nanoparticles, liposomes, gelatin nanoparticles, and silica nanoparticles) into mice [137]. Preclinical results in these four adjuvants showed hollow microneedle delivery induced a strong humoral immune response, with PLGA nanoparticles and liposomes inducing stronger IgG2a response than gelatin nanoparticles and silica nanoparticles assessed as part of this study [137]. A recent clinical study used intradermal vaccination for the treatment of influenza using the Medicago H5-VLP antigen identified from the pathogenic influenza strain, H5N1 [55]. This Medicago H5-VLP antigen was co-administered with the TLR4 GLA adjuvant as part of an aqueous formulation (GLA-AF) [55]. Pre-clinical research was conducted in mice, guinea pigs and ferrets, with each in vivo trial having a distinct purpose. The first study (mice) assessed the immunogenic potential where the adjuvanted groups induced increased IgG2c levels compared with the control (un-adjuvanted) groups. Here, the GLA adjuvant was shown to induce a Th1-biased immune response [55]. On top of this, bone-marrow-derived plasma cells were only detected in the GLA-AF-vaccinated mice, suggesting the generation of a longer-lasting immune response when GLA was present [55].

The second microneedle study (guinea pigs) assessed the safety of the vaccine, where changes in skin condition, weight and body temperature were the three main elements investigated. Here, neither the test nor control animals indicated any safety concerns [55]. The third, final study investigated the protective efficacy in a ferret challenge model to determine function and necessity of the GLA adjuvant [55]. The influenza vaccine (Medicago H5-VLP antigen + GLA-AF adjuvant) aimed to stimulate a rapid response to a pandemic threat, and hence protective efficacy was tested following a single dose of the vaccine [55]. Here, the GLA-AF-adjuvanted vaccine provided an 80% survival rate 3 weeks post challenge with the heterologous A/Vietnam/1203/04 H5N1 strain of influenza virus when compared to the control animals without GLA-AF present [55]. These pre-clinical studies offered sufficient evidence for the commencement of a multi-dose Phase 1 clinical trial (NCT01657929; Table 2) consisting of 105 volunteer patients across multiple centers. This trial assessed safety, immunogenicity and tolerability, including high hemagglutination inhibition (HAI) titres, seroconversion and seroprotection [55]. The immunogenicity data showed that the GLA-AF-adjuvanted vaccine had a higher hemagglutination inhibition titre than the antigen alone. Interestingly, the vaccine response was not statistically significant between the intradermal and intramuscular routes of administration [55]. As expected, the intradermally vaccinated participants experienced transient erythema, but no serious adverse events occurred [55]. The most common adverse effects reported within the intramuscular group were pain, tenderness, erythema at the injection site, fatigue, and mild headaches [55].

GLA currently is formulated with other adjuvanting components (e.g., stable emulsion or alum salts) to improve vaccine compatibility as combination adjuvants against influenza [135]. Except for aqueous formulations comprised of dipalmitoyl phosphatidylcholine (DPPC) based micelles (GLA-AF), the GLA-SE adjuvant (GLA formulated with stable emulsion) is part of an approved seasonal vaccine, Fluzone^®^, where it enhances immunogenicity compared with Fluzone^®^ alone against H1N1 and H3N2 influenza [135,140]. Moreover, the inactivated quadrivalent split-virus influenza vaccine (including haemagglutinin from each of the four influenza virus strains) adjuvanted with GLA-SE produced more peripheral blood mononuclear cells following intramuscular administration into elderly individuals and enhanced Th1 immune responses, overcoming immunosenescence in the elderly [135,141]. This highlights the advantages of combination adjuvants containing GLA, improving both immunogenicity and stability of potential vaccine antigens [135].

### 4.3. Cholera Toxin Adjuvant

Cholera toxin (often abbreviated as CT, Ctx or CTX), is a pathogenic protein complex secreted by *Vibrio cholerae* bacterium, where CT causes diarrhea in infected patients [142]. In recent decades, CT is a powerful mucosal adjuvant has been shown to improve B and T cell response of vaccine antigens [143]. CT consists of two main proteins: subunit A (CTA; a single protein chain expressed as a globular structure with a protruding C-terminal alpha helical extension) and subunit B (CTB; a pentameric structure connected by five CTB monomers where each monomer is linked with two neighboring molecules via hydrogen bonds and salt bridges) [144]. CTA is proteolytically cleaved into two subunits (CTA1 and CTA2) that secrete CT, leading to cholera infection/symptoms [144]. CTB, a non-toxic subunit, is considered a potential adjuvant in vaccine development due to the cellular distribution of its receptor [144]. The adjuvanticity of CT results from its ability to interact with different cell types and bind epithelial cells and increase the permeability of the mucosal barrier, allowing CT and co-delivered antigens to cross the mucosal barrier and induce the maturation of dendritic cells, priming naïve T lymphocytes [143]. CT adjuvants mature human dendritic cells due to CD40 ligand activated dendritic cells. Furthermore, CT adjuvants are enabled to inhibit the production of Interleukin-12 (IL-12), tumor necrosis factor-α (TNF-α), C-C Motif ligand-5 (also known as RANTES), Macrophage inflammatory protein-1 alpha (MIP-1α), and MIP-1β via lipopolysaccharides, as the second pathway to induce the maturation of dendritic cells [143]. Snider et al. indicated that a potential disadvantage of the use of CT adjuvants could stimulate an unwanted immune response to bystander antigens [145,146].

#### Pre-Clinical Development of Cholera Toxin Adjuvants

CT, primarily used as a mucosal adjuvant (Table 1), leads to maturation of dendritic cells, induction of Th2-associated cytokines and antibodies, and induction (or inhibition) of a Th1 response. Th17 cells are a recently identified subset of CD4 T cells, where Th17 cells secrete IL-17, which plays an important role in autoimmunity and the control of extracellular pathogens at mucosal sites [147]. Datta and co-workers hypothesised that Th17 cells contribute to the mucosal CT adjuvant against infectious diseases [147]. Here, Datta’s group combined inactivated bacterial spores derived from the bacillus anthrax (containing or lacking the gene encoding the anthrax protective antigen) with a mucosal CT adjuvant as a vaccine candidate against inhalation anthrax. Mice were intranasally administrated with inactivated bacterial spores combined with PBS, ovalbumin (OVA) or CT plus OVA (CT + OVA) [147]. This investigation indicated that both mucosal IgA and systemic IgA and IgG1 antibodies were present in CT + OVA-vaccinated mice when compared with control groups, against anthrax inhalation [147]. *Naegleria foeleri*, a free-living, thermophilic amoeba ubiquitous in the environment, elicits primary amoebic meningoencephalitis (PAM), a rare and fatal infection in the central nervous system. The first case of PAM occurred in the 1960s in South Australia, with more case reports of PAM now found on a global scale [148]. The understanding and diagnosis of this fatal disease is poor and limited, with no available vaccines against PAM infection currently available [148]. Lee et al. assessed an oral vaccine comprised of the recombinant *Naegleria fowleri* (rNfa1) protein co-administered with CTB to fight against amoeba *N. fowleri* infection and fatal amoebic meningoencephalitis [56]. Pre-clinical assessment was conducted in a murine model and following intranasal administration, enhanced IgG and IgA titres and mice survival rates (100%) from the CTB-adjuvanted vaccines were reported when compared to mice immunised with Nfa1 alone (0% survival). However, additional studies are required to determine the antigen presentation pathways, immune status of mice and protection against *N. fowleri* infection.

Malaria, one of the most common and severe infectious diseases worldwide, elicits high mortality and morbidity especially in low- and middle-income areas, with more than 500 million new cases of malaria reported each year and an estimated 1.1 million deaths [57]. Antibiotics, chemotherapy and physical protection methods (e.g., insecticide-treated bed nets) have made a significant reduction in this malaria epidemic, however, a malaria vaccine is an important and useful method for elimination of this deadly disease [57]. With more than 100 Plasmodium species, high mortality is caused by five primary species (*P. falciparum*, *P. malariae*, *P. ovale*, *P. vivax*, and *P. knowlesi*) [57]. *P. vivax* is the leading cause of malaria-associated morbidity and the primary cause of recurrent malaria, where *P. falciparum* and *P. vivax* are the primary pathogens employed for the development of malaria vaccines [57].

Miyata and co-workers physically combined a *P. vivax* malaria ookinete surface protein (Pvs25) with the CTB mucosal adjuvant as a transmission blocking vaccine (AdPvs25) against malaria [57]. In this pre-clinical study, the AdPvs25 vaccine was subcutaneously, intramuscularly or intranasally administered to mice and provided evidence for CTB-adjuvanted vaccines to reduce parasite transmission to mosquitoes, enhancing a stronger immunogenicity in both subcutaneous and intranasal administration when compared with the un-adjuvanted vaccine [57].

Interestingly, CTB co-administered with other adjuvants (e.g., IFA, cytokines) induced a stronger immune response against the infectious disease antigen. For example, CTB and plasmid-encoded IL-12 were combined with an HIV pathogenic protein (HIV Env protein). Following intranasal administration into mice, the adjuvanted vaccine led to an induction of potent and elevated HIV-specific CD8 responses with protective capacity compared with the CTB adjuvant alone [58]. Although potential use of CT as a mucosal vaccine adjuvant has been documented in a variety of animal models, native CT is highly toxic as a mucosal adjuvant in humans. Further, in mice, cyclic dinucleotides (CDNs) appear to be a safer mucosal adjuvant than CT, promoting protective immunity against H5N1 influenza, *Staphylococcus*, Streptococcus and *Klebsiella* infections [149].

### 4.4. Bacillus Calmette–Guérin

Bacillus Calmette–Guérin (BCG) is an attenuated strain of *Mycobacterium bovis,* with its discovery and development traced back to more than a hundred years ago [62]. *Mycobacteria* species, including *M. tuberculosis* and *M. bovis*, are collectively named tubercle bacilli, and are the cause of severe tuberculosis (TB) in mammalian hosts [150].

In 1908, Albert Calmette and Camille Guérin isolated a virulent strain of *M. bovis* from a cow with tuberculous mastitis [150]. They went on to culture the virulent bovine-type tubercle bacilli in cow bile–potato medium, and after 13 years and over 200 passages, experiments in guinea pigs demonstrated that the virulent tubercle bacillus was eventually attenuated to a stable, non-virulent form (now more commonly known as Bacillus Calmette–Guérin, BCG) [150]. BCG, as an adjuvant, enhances the non-specific immune responses providing protection, with BCG widely used in prophylactic pre-clinical and clinical vaccine development against infectious diseases (e.g., TB, pneumonia, COVID-19) [151].

#### Pre-Clinical and Clinical Development of Bacillus Calmette–Guérin

Prophylactic BCG-adjuvanted vaccines against TB show cellular immune responses required to control tuberculosis, allowing for secretion of systemic memory responses in lung tissue to prevent future TB infections (Table 2) [59]. Intradermal (and intramuscular) administration of BCG does not elicit strong memory responses in the lungs, with Darrah et al. showing intravenous administration of a high dose of a BCG vaccine in rhesus macaques leading to significant increase in antigen-responsive T cells, with a stronger protection against TB infection [59].

A Phase 1b clinical trial (NCT02378207) of BCG vaccination (with recombinant protein vaccines, H4:IC31 and H56:IC31) and BCG re-vaccination was conducted in 481 adolescents where two doses of H4:IC31 and H56:IC31 vaccines were administered intramuscularly 56 days apart, and a single dose of BCG was administered intradermally (Table 2) [61]. This clinical trial demonstrated that the three TB vaccines were safe and tolerated, with no severe side effects reported. The H4:IC31 and H56:IC31 vaccines both induced CD4^+^ T cell immune responses with secretion of IgG antibodies, which were shown to bind to the TB antigen [61]. Further, BCG re-vaccination stimulated potent and polyfunctional BCG-specific CD4^+^ immune responses without IgG binding antigens, and reduced CD8^+^ T cell immunity occurring in clinical trial groups [61]. A Phase 2 clinical trial (NCT02075203) involving 990 previously BCG-vaccinated adolescents showed that BCG re-vaccination was necessary for the prevention of TB infection in un-infected populations (Table 2) [60].

BCG was also assessed as an adjuvant combined with the COVID-19 pathogenic antigen (SARS-CoV-2 derived peptide from non-structural protein 3), to activate the adaptive immune response and provide lasting protection [62,152]. Many nations (e.g., Germany, Finland, and Belgium) mandate BCG vaccination (a live attenuated strain of *M. Bovis*) to newborns due to its effectiveness against TB and leprosy [153]. Here, Escobar and colleagues found a negative correlation between BCG vaccination and COVID-19 mortality [63]. It was reported that BCG as an adjuvant induced human CD4^+^ and CD8^+^ T-cell reactivity to their corresponding COVID-19-derived peptide. Additionally, BCG vaccination increased interferon-gamma production and enhanced non-specific human immune responses through adjuvant effect could be harnessed as cross-protection against the COVID-19 epidemic [63,152]. Phase 3 clinical trials of the SARS-CoV-2 envelop protein adjuvanted with BCG (COVID19-BCG) was conducted in 1120 healthcare workers in France, leading to a reduction in COVID-19 infection compared with the placebo-vaccinated group (NCT04384549; Table 2) [62,153]. Moreover, a randomised Phase 3 clinical trial of this COVID-19-BCG vaccine in healthcare workers was carried out in Holland (NCT04328441) and Australia (NCT04327206), where participants (1500 and 10,078, respectively) also showed significant protection from COVID-19 infection when compared with the placebo-vaccinated group [63].

Although BCG is yet to be recognised as a commercial vaccine adjuvant, its potential as a leading adjuvant for protein-based vaccines is promising given recent investigations into its use as a vaccine adjuvant against COVID-19 infection.

## 5. Emulsions Adjuvants

Water-in-oil (and oil-in-water) are two subtypes of emulsion-based adjuvants, where the oily phase components of these mixtures are derived squalene (sourced from shark liver or plants, including rice bran, olives, and wheat germ) [12]. These adjuvants induce a depot effect with sustained antigen release, and common adjuvants in this class include complete Freund’s adjuvant (CFA), incomplete Freund’s adjuvant (IFA) and montanides (e.g., MF59^®^, AS03 and glucopyranosyl lipid adjuvant-stable emulsion ([GLA-SE])). Both MF59^®^ and AS03 are water-in-oil emulsion systems, with GLA-SE being an oil-in-water emulsion [34,154].

### 5.1. Complete and Incomplete Freund’s Adjuvants

The incorporation of heat-killed *M. tuberculosis*, *M. butyricum* or their extracts (which aggregate macrophages at the inoculation site) with mineral oil and the surfactant mannide monooleate make up a CFA emulsion [155]. The depot effect observed with CFA-adjuvanted vaccines promotes a slow antigen release within the immunised host for up to six months [12]. The CFA adjuvant elicits cellular and humoral immunity via the activation of TLR2, 4 and 9 [155].

In some instances, CFA induces excessive inflammation at the injection site, with reports of immunological toxicity due to the non-metabolisable mineral oil [12]. Thus, at present, CFA is not available for human use, although studies on the activation of CFA provide reliable outcomes for clinical vaccine development [155].

IFA has the same formulation as CFA with the removal of *Mycobacterium* [12]. IFA was designed to minimise the excessive inflammatory effects observed with CFA, and as a result, IFA adjuvants have been (and continue to be) used in veterinary vaccines [156]. However, for human vaccines, a range of serious side effects (e.g., persistent painful granulomas and sterile abscesses at the injection site) in clinical trials of influenza, tetanus and cholera vaccines have been reported when IFA was used (Table 2), limiting its application in human-based vaccine development against infectious diseases [113].

Water-in-oil emulsions (e.g., IFA and CFA) carry a high risk of adverse effects from the use of non-metabolisable mineral oil, which is not licensed in vaccines. Freund’s adjuvants induce strong adaptive immunity, but are so reactogenic that their use even in laboratory animals is discouraged [157].

#### Pre-Clinical and Clinical Development of Freund’s Adjuvants

CFA has been commonly used as an effective adjuvant for experimental antibody production, where its general immunostimulatory abilities have not been replaced by any other adjuvants [158]. However, various lesions of CFA, including injection-site granulomas, spinal cord compression, necrotising dermatitis, and renal granuloma formation, have led to significant limitations for the use of CFA (and IFA) in clinical trials [158].

IFA has been broadly tested in humans, where it enhanced the potent humoral immune responses to a greater extent than commercial adjuvants (e.g., alum and MF59). IFA, used in conjugation with other adjuvants (e.g., saponins or cytokines), leads to potent adaptive immune responses in animal models and may soon be tested in clinical research [156]. The toxicity of IFA is controlled by the use of high-grade oils and purified surfactants, and as a result, IFA has appeared in recent clinical trials for infectious diseases (e.g., HIV; Table 2) [156]. In recent HIV vaccine development, Levine and co-workers performed clinical studies of a HIV-1 immunogen emulsified in IFA on 25 participants (NCT00381875) [64]. This HIV-1 vaccine showed no significant adverse effects in humans following immunisation, and interestingly, participants were followed over a 6-year period, with 12 (of the 25 participants) maintaining a higher level of antibody response [64]. Determination of clinical efficacy of this HIV immunogen as well as significant correlation between HIV-delayed type hypersensitivity response and a more favourable clinical course must be considered [64].

### 5.2. Montanides

Montanides are water-in-oil emulsions comprised of squalene and stabilised with surfactants (e.g., mannide monooleate surfactant) [159]. Montanide adjuvants effectively induce the secretion of Th1/Th2 cytokines to stimulate both humoral and cellular responses [12]. Meanwhile, a depot effect, induced activation of antigen-presenting cells, and improved antigen uptake via interaction with cell membranes, enable montanides to stimulate higher immune responses compared to alum-based adjuvants [160].

#### Pre-Clinical and Clinical Development of Montanide Adjuvants

Montanide ISA-51, an emulsion adjuvant, is formed by the combination of mannide monooleate family with mineral oil [159]. ISA-51 enhances antigen-specific antibody titres and cytotoxic T lymphocyte (CTL) responses in clinical studies [159]. The ISA-51 adjuvant slowly releases antigens at the immunisation site, stimulating antigen-presenting cell uptake and enhancing the accumulation of lymphocytes in lymph nodes [159]. The emulsion-adjuvanted anopheles gambiae saliva vaccine (AGS-v) is a subunit vaccine composed of four different *Anopheles gambiae* saliva proteins adjuvanted with ISA-51 to fight against this mosquito-borne disease [65]. A Phase 1 clinical trial of the AGS-v vaccine was conducted in 49 healthy volunteers (NCT03055000; Table 2), showing that the adjuvanted vaccine stimulated immune responses faster and with increased potency when compared to the antigen (AGS-v) alone. On a side note, the ISA-51 vaccine was reported to have caused more localised side effects at the side of injection (e.g., redness, swelling, itching) when compared with the un-adjuvanted vaccine, with no other serious safety concerns reported [65]. This study provided the first evidence for the efficacy and safety of montanide ISA-51 in saliva protein vaccines, leading to an increase in its use in vaccine development in years to come.

### 5.3. MF59^®^

**Figure 3:**
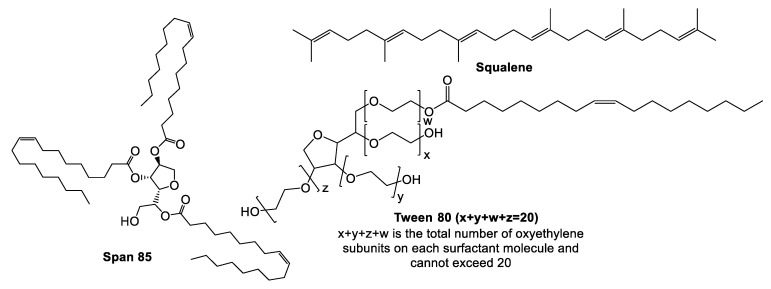
Chemical components of MF59^®^, where MF59^®^ is composed of squalene and two surfactants (Span 85 and Tween 80). MF59^®^ and AS03 not only have similar components, but also analogous composition. These are mixed in an oil phase giving oil-in-water emulsions.

MF59^®^ (Figure 3) is an oil-in-water emulsion comprised of squalene oil (a precursor to cholesterol) and was the first oil-in-water adjuvant to be approved by the FDA as part of an influenza vaccine clinical trial in 1992 [161]. MF59^®^ was first licensed in Italy in 1997 and is now widely approved around the world as a vaccine adjuvant against influenza (e.g., seasonal pandemic and avian influenza vaccines) [66,162].

To prepare an emulsion, lipophilic and hydrophilic emulsifiers (specific formulations) are dissolved in oil and water phases, respectively, where crude emulsions are formed from mixing the two phases with a very high stir rate (e.g., high-shear mixer) [163]. After applying high pressure (e.g., high pressure homogeniser) or strong sonication to the crude emulsion, a balanced emulsion is formed via filtration [163].

The MF59^®^ adjuvant activates monocytes, macrophages and dendritic cells in muscle enabling secretion of various chemokines, leading to a significant migration of immune cells at the injection site [161]. Here, MF59^®^ recruits antigen-specific antibodies, stimulating both Th1 and Th2 immune responses [34]. The indirect depot effect of MF59^®^ allows for prolonged immune cell response and long antibody secretion [34]. Consequently, emulsion-based delivery systems have had a strong developmental history following the discovery of squalene, which facilitated the creation of MF59^®^ and AS03 (approved in H5N1 and H1N1 vaccines), paving the way for application in other infectious disease vaccines [164].

#### Pre-Clinical and Clinical Development of MF59^®^

MF59^®^ was approved in human influenza vaccines, which have been used in more than 30 countries worldwide [161]. The use of MF59^®^ has been particularly prominent in influenza (Novartis) and H1N1 pandemic influenza vaccines (Focetria^®^ and Celtura^®^) [165]. Although MF59^®^ generally has a high safety record, the adjuvant’s effect in vaccines for adults >65 years prompted more stringent, targeted testing due to the weaker immune system of the elderly population [161]. Subsequently, a statistical analysis was conducted by Kelly Lindert confirming the safety of the MF59-adjuvanted trivalent influenza vaccine (aIIV3; Fluad^®^) (NCT04576702) in elderly patients [66].

MF59^®^ has also adjuvanted a SARS-CoV-2 sclamp antigen assessed in Phase I clinical trials (NCT04495933; Table 2) [67]. Here, the MF59^®^-adjuvanted-SARS-CoV-2 sclamp vaccine was found to be safe and well tolerated, where two doses of the vaccine induced a strong SARS-CoV-2 specific polyfunctional CD4^+^ T cell response, with the biased Th1 profile [67].

Squalene oil is prominent in oil-in-water emulsions; however, its immunological mechanism around structure/function of the oil composition are not clear [166]. Further, as the primary source of squalene is from the liver of sharks, this limits its manufacture on a large scale [167]. Emulsified oils from Pinaceae (pine trees) provide a potentially effective supplementary option, and have been recently assessed as emulsified oil vaccines in mice, pigs and ferrets against pandemic influenza [166].

Although MF59^®^ is an approved adjuvant in infectious disease vaccines, the side effects from this adjuvant still warrant consideration and testing for new vaccines. Further, understanding the mechanism of emulsion-based adjuvants is expected to extend the use of these systems.

### 5.4. GLA-SE Adjuvant

GLA-SE contains the TLR4 agonist glucopyranosyl lipid adjuvant (GLA) and a stable emulsion nano-emulsion of squalene oil-in-water (SE). GLA-SE induces potent Th1 and balanced IgG1/IgG2 responses against various infectious diseases (e.g., tuberculosis, leishmaniasis, or *Mycobacterium leprae* infection) [168]. The addition of GLA-SE also produced the magnitude and polyfunctional cytokines or/and chemokines from CD4^+^ T cells (e.g., TNF-α, IL-6, and IL-12) with acceptable safety and tolerance [69].

#### Pre-Clinical and Clinical Development of GLA-SE

GLA-SE has been extensively used in vaccine development for the past three decades [169]. Respiratory syncytial virus (RSV) is a fatal disease in the elder generation, and to date, there is no approved vaccine [170]. Patton and colleagues evaluated the immunogenicity of an RSA vaccine containing selected antigens from the RSV-soluble fusion protein and combined them with GLA-SE (Table 2). Following immunisation in cynomolgus macaques, GLA-SE-adjuvanted RSV antigens stimulated stronger and longer-lasting humoral and Th1-biased cellular immune responses when compared to an un-adjuvanted RSV vaccine [170].

Cauwelaert et al. demonstrated that this GLS-SE adjuvant stimulated an innate immune response, resulting in IFN-γ expression by NK cells and memory CD8^+^ cells and the stimulation of Th1 immune response [168]. GLA-SE also enhanced the adaptive immune response leading to tuberculosis protection [168].

Schistosomiasis (also known as bilharzia or snail fever) is a disease caused by parasitic worms and infects people who live in tropical climates. Schistosomiasis is second only to malaria as the most devastating parasitic disease worldwide, with more than 800 million people at risk and approximately 200 million infected in 74 countries [68]. An anti-schistosomiasis vaccine entered Phase 1 clinical trials in Brazil using the Sm14 antigen (from the *S. mansoni* pathogenic protein) adjuvanted with GLA-SE (NCT01154049; Table 2) [68]. This vaccine stimulated enhanced anti-Sm14 IgG antibody response with no adverse side effects (aside from mild pain and redness at the injection site) compared to the placebo groups [68].

Further, Phase 1 clinical trials of a TB vaccine (NCT01599897) comprised of *M. tuberculosis* protein (ID93) adjuvanted with GLA-SE. Following immunisation into 60 healthy volunteers, the ID63-GLA-SE vaccine produced higher titres of ID93-specific antibodies (IgG and Ig4 subclasses) and stimulated a more robust Th1 type cellular and humoral response, with a larger magnitude of polyfunctional CD4^+^ T cell cytokines compared with ID93 alone vaccine [69].

### 5.5. TiterMAX Adjuvants

TiterMax adjuvants (TiterMax^®^ and TiterMax Gold^®^) contain a metabolisable squalene oil, sorbitan monooleate 80, non-ionic block co-polymers (CRL8941 and CRL8300), and microparticulate silica [158]. TiterMax^®^ adjuvants, typical water-in-oil emulsions, are less toxic than CFA and contain no biological materials [12]. Non-ionic block co-polymers consist of linear chains of hydrophilic polyoxyethylene and flank linear chains of hydrophobic polyoxpropylene, which secrete complement immune cells and enhance the expression of MHC-II on macrophages, leading to a higher level of IgG1 and IgG2a antibody immune responses [12]. TiterMax Gold^®^ is considered as an improved version of the traditional TiterMax adjuvant [12]. TiterMax Gold^®^ is an effective adjuvant to enhance cellular and humoral immune response in animal studies, as an alternative to Complete Freund’s Adjuvant due to safer and easier use. The immunol potency of the TiterMax Gold^®^ adjuvant depends on a stable water-in-oil emulsion, which capsulates into effective antigens derived from pathogens [171]. The TiterMax adjuvant is often used as an alternative to CFA since it produces a similar antibody titre, although TiterMax has been shown to induce immunisation-elicited lesions [158]. For example, Leenaars and co-workers demonstrated that intraperitoneal immunisation of TiterMax induced severe lesions, with only minimal lesions (and low immunogenicity) found following subcutaneous immunisation with the same vaccine adjuvant, significantly limiting the use of TiterMax as an adjuvant in clinical research [158,172].

#### Pre-Clinical Development of TiterMAX Adjuvants

TiterMax^®^ and TiterMax Gold^®^ are an alternative to Freund’s Complete adjuvant in animal research based on their safety and efficacy. Henker et al. subcutaneously immunized rats with recombinant myosin regulatory light chain capsulated into TiterMax Gold^®^ adjuvant. This study indicated the significant reduction in fluke burdens of 61.5% (*p* < 0.001) compared with the no adjuvanted groups, with mixed Th1/Th2 immune responses [171]. Further, assessment of TiterMax^®^ adjuvant as part of an anti-Schistosoma vaccine by Leite and colleagues indicated a stronger recruitment of mast cells and basophils produced, leading to more IgG2a antibodies [12].

### 5.6. RIBI Adjuvant

Unlike Freund’s adjuvants, RiBi adjuvant systems (RAS) are oil-in-water adjuvants that have been studied since 1985 [158]. This RAS adjuvant system is comprised of a metabolisable squalene oil, immunostimulators and Tween 80 surfactant, where antigens are encapsulated by oil before emulsification in water [158]. RAS oil-in-water emulsions are easily sterilised by filtration, with three immune-stimulators commonly used in RIBI adjuvant systems: trehalose 6,6′-dimycolate (TDM), cell wall skeleton (CSW) and monophosphoryl lipid A (MPL).

TDM is the lipid component of mycobacterial cord factor isolated from virulent strains of *Mycobacteria*, and known to induce both humoral and cellular immune responses post vaccination [158]. CSW is extracted from the mycobacterial cell wall, where the strong immunogenicity of CSW is related to the polymerised form of muramyl dipeptide (MDP), derived from mycobacterial cells as an immunoreactive peptide [158,173]. MDP enhanced antibody production, increased non-specific immune response to pathogens (including bacteria, fungi, viruses, and parasites) and cytokine release, with the induction of autoimmunity and cell-mediated immunity [174,175]. MPL, a TLR4 agonist that induces both B cell and T cell immune responses, is a chemically modified from lipid A that has reduced cellular toxicity [158]. However, the RIBI adjuvant system is known to cause pathological lesions in animals, demonstrated in 1991 by Johnston et.al, where it was found that RIBI caused the same level of lesions as CFA in rabbits [158]. Hence, RIBI adjuvant is approved for use in animal studies for vaccine development against diseases, instead of clinical trials [158].

#### Pre-Clinical Development of RIBI Adjuvant System

Pre-clinical assessment of the immunogenicity of RASs were evaluated in *Mycobacterium paratuberculosis* (MPT) by Mullerad and colleagues. Here, they overexpressed and purified the 85B antigen of MPT and combined it with the RAS before administering to mice [71]. The result of this pre-clinical trial showed that the RIBI adjuvant produced a higher level of IL-10, nitric oxide and the secretion of IgG1 and IgG2a antibodies, when compared with the adjuvant-free vaccine [71]. Cargnelutti and co-workers developed recombinant viral nucleoprotein (NP) as the primary pathogenic for the influenza epidemic, and adjuvanted it with RAS [70]. Following murine vaccination, a higher level of IFN-γ, IgG2a antibodies and the induction of a more robust humoral and cell-mediated immune response was observed by the NP-RAS vaccine when compared to the un-adjuvanted NP vaccine [70].

Preclinical studies that have compared and evaluated the pathological lesions induced by RIBI adjuvant have yielded various results. Johnston and colleagues (1991) and Leenaars and co-workers (1994) found lesions comparable to those induced by CFA in rabbits, whereas others have noted only minimal lesions when using RIBI as an adjuvant [172,176]. Studies comparing intraperitoneal CFA and RIBI in mice consistently report fewer pathological lesions with RIBI [158,177]. With the exception of the report of Lipman’s group in mice, studies comparing the antibody response between RIBI and CFA/IFA have generally indicated a much higher antibody production with the CFA/IFA regimen [158].

## 6. Immunostimulatory Complexes

### 6.1. Cytokines

Non-toxic adjuvants enhance the immune response against specific diseases [178]. Cytokines, as a type of non-toxic adjuvant, affect the functional diversity of immune responses and play a significant role between antibody and cell-mediated immune response [179]. Cytokines, small proteins released by cells, directly affect the interactions and communications between cells [180]. Cytokines are a broad and loose category name for these small and important proteins, where other names include: lymphokines (cytokines made by lymphocytes), monokines (cytokines secreted from monocytes), chemokines (cytokines with chemotactic activities), and interleukins (IL) (cytokines produced by one leukocyte and acting on other leukocytes) [180]. Cytokines may act on the cells that secrete them (autocrine action), on nearby cells (paracrine action), or in some instances on distant cells (endocrine action), where similar functions are induced by different cytokines due to their redundancy [180].

Typically, one cytokine activates its target cell(s), leading to the secretion of additional cytokines and a common cascade [180]. As multiple cytokines are responsible for the induction of innate and adaptive immune responses, this affects the maturation of antigen-presenting cells, differentiation of Th1 and Th2 cells, and secretion of cytotoxic natural killer cells and CTLs, eventually forming the effective protective layers against invasive infections [181]. Thus, several cytokines (including granulocyte-macrophage colony stimulating factor [GM-CSF], INFs, IL-1, IL-2, IL-6, IL-12, IL-15, IL-18, and chemokines) have huge potential for acting as vaccine adjuvants for the stimulation of potent immune responses against infection, particularly in DNA vaccines. Plasmid DNA vaccines contain a gene encoding the desired antigen with respective epitopes that stimulate adaptive immunity (but with poor immunogenicity) [182]. However, commercial and traditional vaccine adjuvants cannot enhance the required immunogenicity of DNA vaccines [182]. Hence, molecular adjuvants (e.g., cytokines, chemokines, and immune-targeting genes) which are plasmid-encoded proteins, have been assessed as vaccine adjuvants. These target innate immune receptors or regulate signal recognitions [183]. The moderate secretion of the recombinant cytokines via infected cells at the injection site effectively limits adverse effects of cytokines [182]. Cytokine-encoding genes are delivered as a separate plasmid, as well as additional genes encoded within specific antigens comprised of the plasmid against the infection (e.g., HIV, influenza, and SARS-CoV) [184].

GM-CSF directly affects the stimulation of dendritic cell formation, resulting in successful antigen uptake, production of CD4^+^ and/or CD8^+^ T cells, and stimulation the specific adaptive immune responses against infection [185].

Interferons (INFs) adjuvant were first discovered as an antiviral factor that interferes with viral replication in mammalian cells. Infected cells and active innate immune response can secrete interferons, with cytokine production as well as the induction of natural killer cell functions and antigen presentation. INFs are classified into three different types (Type I, II, and III) based on their structural homology and related specific receptors [186]. Type I IFNs induce apoptosis of invasive viruses, enhance cross priming and the presentation of viral peptides [187]. Type I IFNs, as B cell activators, secrete various antibodies and induce a strong humoral immune response via intranasal (or intramuscular) administration [187]. T lymphocytes, natural killer cells (NK) and natural killer T (NKT) cells produce type 2 INFs, which have antiviral, antitumor and immunoregulatory functions [188]. This type 2 INF cytokine commonly controls the differentiation of macrophages, MHC expression in antigen-presenting cells, and the maturation of Th1 (CD4^+^) and cytotoxic CD8^+^ cells [188]. Furthermore, immunoglobulin class switching is regulated by type 2 INF [188].

Interleukin (IL) family cytokines (e.g., IL-1, IL-2, IL-12, IL-15, and IL-18) are significantly involved in adjuvant development. IL-1 is an ancient and significant pro-inflammatory cytokine leading to potent effects on the hosts immune system [189]. IL-1 enhances the activity of cells (neutrophils, eosinophils, basophils, mast cells, and NK cells) required for an innate immune response, activating and reinforcing the function of polarised T cells [189].

IL-2, primarily produced by CD4^+^ T cells, affects the proliferation and induction of T cells with the stimulation of humoral and cellular immune responses [190]. IL-2 is involved in the expression of CD48 and CD80 on dendritic cells, as well as upregulation of their respective ligands (CD2 and CD28) on CD4^+^ T cells [190].

IL-12, a Th1 pro-inflammatory cytokine, is produced by phagocytes and dendritic cells in response to infections by pathogenic microbes [190]. IL-12 enhances the production of NK, T and B cells, and regulates the differentiation of Th1 cells [190].

IL-15 is produced by monocytes, dendritic cells, epithelial cells, bone marrow stromal cells, and fibroblasts, with high homology and structural similarities with IL-12 [190,191]. IL-15 regulates the secretion and proliferation of NK and T cells, as well as B cell antibody production [190].

IL-18, produced by macrophages and Kupffer cells, regulates the secretion of IL-2 and IL-12, and enhances the proliferation and activity of NK and CD8^+^ T cells [190]. IL-18, as a primary promoter of a Th1 immune response, affects the differentiation of Th1 immune cells and the production of IFN-γ [190].

#### Pre-Clinical and Clinical Development of Cytokines

GM-CSF is a pleiotropic cytokine, which has been used in studies against infectious diseases for Mycobacterium tuberculosis, Epstein-Barr virus, and human immunodeficiency viruses [74]. Kasahara and co-workers identified that the GM-CSF receptor (a heterodimer consisting of a ligand-binding subunit [GM-CSFRα/Csf2ra]) was a common signal-transduction subunit (GM-CSFRβ/Csf2rb) against *Aspergillus fumigatus* [192]. Here, mice without the GM-CSF receptor B chain expressed invasive hyphal growth and exhibited a reduction in their survival following infection with *A. fumigatu* [192]. However, mice administrated with recombinant GM-CSF enhanced neutrophil NADPH oxidase (catalysing the transfer of electrons to oxygen generating superoxide) function, candidacidal activity and strong fungal clearance [192].

As an adjuvant, GM-CSF was conjugated with a pseudorabies virus (PrV) glycoprotein and administrated to mice as a vaccine. This resulted in an improved production of IgG and stronger T cell-mediated immune responses biased for a Th1 type response, leading to enhanced protective immunity against Prv infections compared to the adjuvant-free vaccine [193]. Furthermore, GM-CSF combined with HIV-1 envelope glycoproteins (Env) was delivered as an HIV vaccine into rhesus macaques [194]. This pre-clinical trial concluded that the conjugation of GM-CSF adjuvant with HIV antigens elicited higher neutralising antibody levels and better control of the primary infection and re-emergent virus than the antigen alone vaccine [194]. Further, a *hepatitis B virus vaccine* (HBV) vaccine was developed by Fabrizi and colleagues using GM-CSF combined with the protein on the surface of HBV and performed meta-analysis in 187 patients. This study demonstrated that this GM-CSF/HBV vaccine significantly improved the sero-protection rate in those patients with end-stage renal disease [195]. Brekke and colleagues combined HIV Vacc-C5, containing residues 489–511 from the HIV-1 virus C5 domain, with GM-CSF adjuvant as a peptide-based therapeutic vaccine against HIV [196]. In this Phase 1/2 clinical study, Vacc-C5 adjuvanted with GM-CSF was intramuscularly administrated to 36 participants and provided evidence for the GM-CSF-adjuvanted vaccine to be safe and well-tolerated in all patients and induce the stronger T cell immune response compared to vaccine alone (NCT01627678) [73]. A Phase 1 clinical trial in progress is assessing a vaccine containing dendritic cells loaded with S-protein from SARS-CoV-2 alongside GM-CSF as an adjuvant intramuscularly injected in 175 non-infected participants to evaluate its safety and anti-SARS-CoV-2 immunity (NCT04386252) [74]. This trial has only just started, and clinical results are pending [74]. As a vaccine adjuvant, GM-CSF enhances the immunogenicity of vaccines to mount a strong immune response. GM-CSF has been tested in both animal and human trials as a vaccine adjuvant against infections as well as anti-tumor immunotherapy in prostate, breast, or lung cancers [74].

Type 1 INF is a common adjuvant used for mucosal vaccination against H1N1 virus, H3N2 virus hepatitis B virus, and influenza B virus. For example, Bracci and colleagues combined inactivated subunit H1N1 influenza protein antigens with type 1 INF, which was prepared in the C243-3 cell line and concentrated and partially purified by ammonium sulphate precipitation and dialysis against phosphate buffered saline (PBS), as an influenza subunit vaccine, which is intranasally delivered into mice [197]. This pre-clinical study demonstrated that this influenza subunit vaccine produced higher antibody titres (IgG2a and IgA) and increased the percentage of antigen-associated phagocytes in the nasal mucus layer, leading to strong humoral protection against influenza challenge with A/New Caledonia/20/1999 (H1N1) influenza virus, when compared with the influenza antigen alone [197]. The adjuvanticity of type 1 IFN enhanced the efficacy of experimental vaccines in mice against influenza viruses, with the production of antibodies and long-term immunological memory [198]. Type 1 IFN adjuvant enabled vaccine-induced humoral immunity via the stimulation of dendritic cells, as well as B and T cells [198]. However, Couch et al. combined inactivated influenza virus vaccine with MPL, type 1 INF or cholera toxin B as influenza vaccine intranasally administrated in healthy adult volunteers. Vaccinations were well tolerated, while type 1 INF did not exhibit an adjuvant effect for the humoral immune response in humans to inactivated influenza virus administrated intranasally [199].

Chronic HBV infection (CHB) a significant global public health problem and the cause of severe diseases (e.g., cirrhosis, liver failure, and hepatocellular carcinoma) affects more than 280 million people with over 800,000 deaths each year [72]. Patients who test positive for more than 6 months following hepatitis B viral infection are diagnosed as having a chronic hepatitis B viral infection, where the human immune system cannot get rid of the virus. There are no vaccines for the prevention and/or treatment of CHB infection. BRII-179, a virus-like particle-based vaccine containing all three HBV surface envelope proteins (Pre-S1, Pre-S2, and S) combined with IFN-α as a CHB vaccine [72]. Phase 1 clinical trial of this CHB vaccine intramuscularly administrated into healthy volunteer aged between 18 and 60 years old (ACTRN12619001210167) has been completed [72]. Here, BRII-179, as a novel formulation protein vaccine comprised of all three HBV surface envelope proteins (Pre-S1, Pre-S2 and S) adjuvanted with IFN-α was safe, well tolerated and induced HBV-specific T cell immune responses, but was of limited efficacy in virally suppressed, non-cirrhotic patients with CHB [72]. A recent Phase 2 clinical trial (NCT04749368) conducted further evaluation of the efficacy of BRII-179 and BRII-179 adjuvanted with INF-α, and another HBV-specific vaccine, BRII-835, as investigational HBV-targeting siRNA sequences adjuvanted with IFN-α, and they were subcutaneously administrated in 135 patients [72]. This Phase 2 clinical trial is expected to release its clinical results in 2023.

Influenza is a viral infection of the respiratory tract mucosa from the nose to the terminal bronchioles, where the majority of antibody secretion occurs at the mucosal surface. Hence, influenza vaccines should induce both a mucosal immune response and Th2 cellular response, with the secretion of antibodies and memory cells. Further, 20 to 99 amino acid strains were selected from influenza A viruses (H1N1 and H3N2) adjuvanted with INF-α were assessed clinically following intranasal immunisation in 90 healthy volunteers aged between 18 to 40 years of age (NCT00436046) [75]. This Phase 1 trial concluded that intranasal administration produced higher levels of IgA and IgG2 antibodies and induced the required protective mucosal immunity when compared to placebo groups [75]. In 1989, Stürchler and co-workers synthesised a malaria sporozoite peptide combined with INF-α adjuvant as a malaria vaccine candidate intramuscularly given to healthy volunteers [200]. This clinical trial indicated that higher antibody titres and the stronger humoral immune response to the sporozoite peptide produced in the INF-α-adjuvanted group in comparison to the placebo group [200]. The amount of antibody needed to generate a protective immune response against sporozoites was unknown, however, the amount of antibodies elicited by IFN-α combined with the sporozoite vaccine has been questioned due to no clear understanding of IFN-α as an adjuvant, indicating significant limitations of this clinical study [200]. The INF-α adjuvant is pleiotropic cytokines with the long-term record of clinical use [201]. INF-α affect innate and adaptive immunity and elicit INF-DC interactions for the stimulation of the adaptive immune response [201]. INF-α adjuvant is the use of IFN-α or IFN-α-conditioned dendritic cells against infections (e.g., malaria, influenza, and HBV) as well as anti-tumor therapy [201].

In addition to this, influenza vaccine research has been the foundation of interleukin research development for IL-1, IL-2, and IL-12. IL-2 is the first adjuvant in the development of influenza vaccine. In 2006, IL-2 was co-delivered with a DNA vaccine against influenza when Henke and co-workers generated an influenza A virus bicistronic plasmid [202]. This DNA vaccine candidate was intranasally injected into mice, leading to an effective prevention of a lethal challenge of influenza virus in comparison to the vaccine administered without the IL-2 adjuvant [202]. Furthermore, IL-2-encoded DNA vaccine plasmids were designed by Hu and co-workers against severe acute respiratory syndrome (SARS) in 2009 [203]. The IL-2 gene as a cytokine-adjuvant-encoded SARS-CoV nucleocapsid DNA vaccine was intramuscularly administrated into mice, leading to higher IgG titres and a more effective specific immune response in comparison to the vaccine antigen administered without the IL-2 adjuvant [203].

A single vaccine has used IL-1 as an adjuvant for infectious disease vaccination. Here, IL-1, another safe and potent adjuvant, has been used in the development of mucosal vaccines against influenza [181]. Kayamuro et al. designed a recombinant influenza virus hemagglutinin conjugate containing IL-1 family cytokines (IL-1α, IL-1β, IL-18, and IL-33), which was intranasally administered into mice [181]. This pre-clinical trial showed that following vaccination, plasma IgG and mucosal IgA significantly increased along with the production of Th1 and Th2 cytokines, leading to a humoral immune response for protection against influenza viruses when compared to non-adjuvanted groups, with no acute toxicity [181]. Specific IL-1cytokines have been used as vaccine adjuvants to induce the strong adaptive and memory immune response against infections (influenza) or cancers, with an effective adjuvant in mucosal vaccine [181]. IL-18 is responsible for the activation of Mast cells and in augmentation of CTL immune response elicited by IL-33 as a mucosal adjuvant [181]. Although IL-1 family cytokines are a potent nasal vaccine adjuvant involved against infectious diseases, the combination of IL-1 cytokines with commercial adjuvant (MF59 or alum adjuvant) is an effective pathway to induce both humoral and mucosal immune response against pathogens entering the mucosal layer [204].

To further adjuvant investigation against influenza, IL-12 (mucosal adjuvant) has also been investigated [205]. Mice were intranasally administrated the influenza vaccine candidate (comprised of soluble hemagglutinin (H1), neuraminidase (N1) and soluble recombinant IL-12 adjuvant), leading to increased levels of IgG2a anti-HIN1 antibody in serum, and IgG1, IgG2a, and IgA antibodies in bronchoalveolar lavage fluids compared to the antigen alone [205]. Moreover, the IL-12 adjuvant remarkably enhanced mice survival, stimulating a stronger protective mucosal immune response [205]. Kumar et al. conjugated IL-12 with inactivated Yersinia pestis CO92 agonist and intranasally delivered into mice [206]. The result showed an enhanced safety, immunogenicity and protective efficacy against pneumonic plague [206]. RNA-optimised HIV-1 gene DNA derived from HIV strain was administrated either intramuscularly alone or in combination with dose-escalation of IL-12 or IL-15 plasmid cytokine adjuvants in healthy volunteers (NCT00111605) [207]. This Phase 1 clinical study showed that vaccination with the HIV immunogen and plasmid cytokine adjuvant was safe and well-tolerated, with no significant adverse events, whereas both IL-12 and IL-15 cytokine adjuvant offered little ability to augment cellular immunity [207]. IL-12 is a typical heterodimeric cytokine, which is secreted by macrophages, dendritic and Langerhans cells, inducing the mucosal and adaptive immune response in mice models against infections (such as influenza or HIV). However, there are very few clinical trials about IL-12 as a vaccine adjuvant against infections due to low immunogenicity [206].

A cytokine-plasmid encoding IL-15 combined with *Trypanosoma cruzi* (the agent of Chagas disease) trans-sialidase (TS) gene plasmin was administrated into mice dramatically improving the long-term protection following secretion of CD8^+^ memory T cells [191]. In addition to this, IL-15 has been assessed as part of a viral-based HIV gp160 vaccine in a murine model [191]. Here, incorporation of IL-15 induced a long-term, antigen-specific CD8^+^ T cell immune response as well as a long and potent antibody-mediated immune response [191].

Lastly, IL-18, known as IFN-γ inducing factor, is a member of the IL-1 cytokine family of ligands [208]. Its initial discovery has been traced back to the last decade and IL-18 appears to contain unique characteristics, some of which can treat sepsis and infections (influenza, brain inflammation, and HSV-1) [208]. For example, IL-18 adjuvanted a herpes simplex virus DNA vaccine (HSV-1) where, following pre-clinical evaluation in a murine model, the IL-18-adjuvanted vaccine strongly enhanced a Th1 immune response with strong protective immunity against HSV-1 infection [209].

Cytokines are a potential adjuvant for vaccines against infectious diseases due to the induction of effective mucosal and memory immunity. Thus, cytokines as a nasal vaccine adjuvant combined with commercial adjuvants (alum, MF59, and AS03) can enhance the potent adaptive immunity, with simple intranasal administration.

### 6.2. Chemokines

Chemokines are a family of small proteins that are best known for their ability to stimulate the migration of cells, where over 50 chemokines (and 18 chemokine receptors) have been identified [76]. These chemokines are classified into four different families (CC, CXC, C, and CX3C) based on the arrangements of the first two conserved cysteine residues [76]. Chemokines affect the proliferation and maturation of lymphocytes with the regulation of the innate and adaptive immune responses, and as a result, chemokines have been utilised as vaccine adjuvants based on their capabilities to affect adaptive and/or protective immune responses [76]. Chemokine adjuvants co-administered with DNA vaccines enhance APC delivery to the injection site where the antigen is up taken, improving the immune response against the infection [183]. However, limitations of chemokine adjuvants around dose-related toxicity issues and stability have been reported [76].

#### Pre-Clinical Development of Chemokines

The CCL3 chemokine attracts NK and CD8^+^ T cells and has been used as an adjuvant for HIV-1 vaccines [76]. Here, Kuczkowska et al. created a truncated HIV-1 Gag antigen with murine chemokine CCL3 as an HIV vaccine, which led to the induction of the strong antibody titres and CD4^+^ T cell responses [76]. In addition to this, CCL28 has been used in influenza vaccines where CCL28 was adjuvanted with influenza virus-like particles and intranasally administrated into mice [76]. This study demonstrated that the CCL28 chemokine enhanced production of IgA (not IgG or IgM), promoting migration of IgA to different mucosal sites, and increased mucosal antibody titres and long-term protective immune response compared to the un-adjuvanted vaccine [76].

RANTES, also known as a CCL5 chemokine, recognises monocytes, memory T cells and eosinophils, inducing both mucosal and systemic immune responses [210]. In 2000, Kim et al. encoded RANTES-expressing plasmid co-administrated with hepatitis B surface antigen (HBsAg) as a DNA vaccine, and intramuscularly (or intravaginally) immunized mice [210]. The results indicated that the intravaginal administration of RANTES-encoding plasmid with HBsAg produced higher levels of both serum IgG and mucosal IgA with a stronger protective immune response, compared to adjuvant with cholera toxin adjuvant [210]. Moreover, in 2001, a macrophage inflammatory protein 2 (MIP-2) plasmid, with a DNA vaccine encoding glycoprotein B of herpes simplex virus (HSV gB) was intranasally administrated into mice, generating stronger IgG immune responses and Th1-like immune responses than the no-mucosal immune responses of MIP-2 DNA vaccine. Macrophage inflammatory protein 2(MIP-2) is a type of α-chemokine with a strong neutrophil chemoattract [211]. Eo and co-workers combined a MIP-2 plasmid with a DNA vaccine encoding glycoprotein B of herpes simplex virus (HSV gB) that was intranasally administrated into mice, generating a higher level of IgG titres and Th1 immune response as compared to DNA vaccine alone, while no mucosal immune response of MIP-2 DNA vaccine [211]. Thymus-derived chemotactic agent 3 (TCA 3) expresses chemotactic activity to monocytes, macrophages, neutrophils, and eosinophils [211,212]. Tsuji and colleagues (1997) co-administrated a plasmid-encoding murine TCA3 with a DNA vaccine expressing HIV-1 that was intramuscularly delivered to mice, resulting in a strong HIV-1 cell-mediated immune response without significant humoral immunity [211,213]. To date, despite these promising pre-clinical results, no further clinical research employing chemokines as infectious disease vaccine adjuvants has been reported.

Chemokines are easily engineered into DNA vaccine constructs or administrated as a separate plasmid, with the enhancement of systemic or mucosal immune response [183]. Hence, compared to cytokines, chemokines are more stable and less toxic, leading to potential as DNA vaccine adjuvants against infectious diseases [183].

## 7. Particulate Adjuvants

### 7.1. Imidazoquinolines

Imidazoquinolines arose from the development of a nucleoside analogue, and despite their molecular structure being similar to nucleosides, imidazoquinolines have a different mechanism associated with nucleoside-like activity [214]. Imidazoquinolines primarily bind with TLR7 (and/or TLR8) to produce type 1 and type 2 INF cytokines IL-12 and TFN-α, respectively resulting in the induction of innate immune responses and potent Th1 biased adaptive immune responses [27,78]. Imidazoquinolines, as small synthetic molecules, are produced cost-effectively with high purity and were first approved by the Food and Drug Administration (FDA) in 2010, as a 5% cream, which is widely used in the treatment of genital warts and skin cancer [214,215]. Unformulated imidazoquinolines (e.g., R848) affect systemic immune activation due to their size and solubility [215]. However, imidazoquinolines as vaccine adjuvants have been involved in clinical trials of cancer therapeutic vaccines, while this adjuvant has not been utilised in human research against infections [215].

#### Pre-Clinical Development of Imidazoquinolines

Imidazoquinolines have been clinically assessed for cancer and bacterial diseases, but not for infectious diseases. The pre-clinical assessment of an intranasal RSV vaccine adjuvanted with a covalently linked imidazoquinolinone delivered into mice demonstrated a higher level of IgG1 antibodies with a robust humoral immune response when the dissemination of inflammation occurred in mice lungs in comparison with un-adjuvanted mice [78].

Resiquimod (R848), an imidazoqiniline, is a dual TLR7 and TLR8 synthetic agonist that has been used as an immune-stimulant in humans against viral diseases [27]. R848 conjugated with inactivated influenza virus (IPR8-R848) showed strong efficacy and safety in a neonate nonhuman primate model, stimulating enhanced virus-specific T cell responses and IgG and IgM antibodies when compared to the un-adjuvanted inactivated viral vaccine [77].

### 7.2. Virus-like Particles and Virosomes Adjuvants

Micro- and nano-particles have been used as adjuvants (or delivery systems) for vaccines, where common ones used in infectious disease research include virus-like particles (VLPs), and virosomes [216]. In these systems, antigens are coated on the surface of (or within) these particles by physical adsorption or chemical bonds.

**Figure 4:**
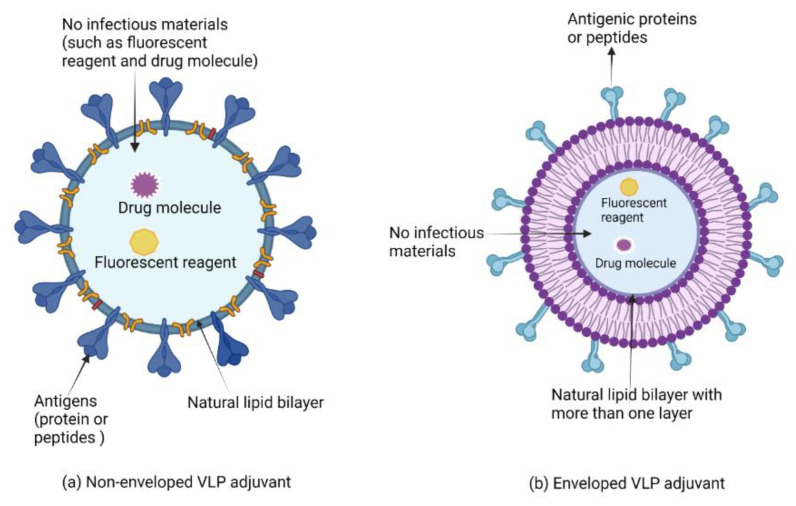
Schematic of a virus-like particle (VLP), where the surface is made up of antigens (e.g., capsid proteins) that have been embedded into the natural lipid bilayer (surrounding cell membrane) to form enveloped VLPs. (**a**) Non-enveloped VLPs contain single or multiple capsid proteins only. Neither type of VLP contains infectious material (e.g., DNA/RNA). (**b**) The formulation of double layer enveloped VLPs is related to the multiple glycoproteins on their surface.

VLPs retain their virus-derived structures with the similar size and form of native virus particles but lack DNA/RNA constituents from the virus itself, disabling their capability to infect the host cell [217]. Icosahedral and rod-shaped structure are in VLPs, which are made by the self-assembly of viral structural proteins [217]. VLPs can be experimentally produced in various expression systems (e.g., mammal cell lines, plants, bacteria, yeast, and insect cell lines), as carriers for the delivery of bio and nanomaterials, including vaccines or drugs by virtue of the cavity within their structure [217]. Here, the morphology of VLPs with self-assembled structural capsid proteins is analogous to native viruses, where antigens are displayed on the outer surface. VLPs, trafficked from the injection site to the lymph nodes, are efficiently taken up by antigen-presenting and dendritic cells, inducing a potent immune response [218]. The repetitive arrangement of antigens on a VLP activates B cells, stimulating a stronger humoral immune response, as well as induced T cell activation and a cellular-mediated immune response [218]. Based on the structure of VLPs, which can be divided into non-enveloped VLPs and enveloped VLPs (eVLPs). Non-enveloped VLPs contain single or multiple-capsid proteins of a specific virus, with a simple and relative structure [217,219]. Enveloped VLPs are significantly complex in structure and consist of both virus and host membrane components [219]. Enveloped VLPs have limited immune stimulatory ability (based on the structural proteins used), functioning more effectively as a delivery system for both antigens and adjuvants, with immunogenic material presented on the outer surface of a VLP (Figure 4), while the lipid bilayer also contains immunogenic particles to enhance antibody response [217]. Several prophylactic vaccines (e.g., Recombinvax HB and Engerix-B for hepatitis B virus, Gardasil, Cervarix, and Gardasil-9 for human papillomavirus, and Hecolin for hepatitis E virus) are non-infected structural proteins from viruses, utilized as VLP-based vaccines, with commercially available on the market [217,219].

Advantages of VLPs as a delivery system for vaccines include specific targeting, effective host-cell penetration, biocompatibility, and degradability [219]. VLPs are similar to natural viruses, and antigens exposed on the surface of VLPs, with effective uptake by antigen-presenting cells and faciliatory of endocytosis and penetration of antigens in the host cells [219]. Additionally, proteolytic mechanisms of VLPs can allow VLP-based vaccines to be simply degraded, and the degradative products are biocompatible [219].

Despite many cellular platforms used to generate VLPs (e.g., insect, mammalian, bacteria, yeast, and plants), VLPs cannot express the essential post-translation modifications for optimal immunogenicity in humans [220]. In bacterial platforms, contamination of endotoxins may occur in VLP formulation. Yeast and insect cells are cost-effective and scalable to form VLPs, while forming unexpected modifications or contaminants, including eukaryotic post-translational modification (e.g., glycosylation) and baculovirus contaminants, respectively [220]. These components or modifications are enabled to negatively affect the stimulation of the adaptive immune response against diseases. Mammalian cells are the most difficult to produce VLPs due to the existence of human viral pathogen contamination and complex post-translation modifications [220].

**Figure 5:**
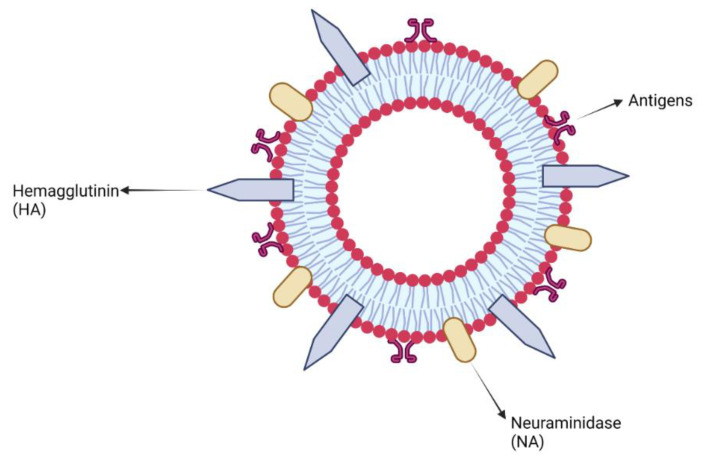
Schematic virosome adjuvant. Virosomes are synthesised from a phospholipid bilayer (similar to that of a liposome) where approved influenza vaccines are used the virosomes virus structure allowing for conjugation with influenza surface protein hemagglutinin (HA) and neuraminidase (NA).

Virosomes, are spherical, unilamellar vesicles (60–200 nm), consisting of viral envelopes phospholipids with removed nucleocapsid (Figure 5) [82].

Virosomes, a type of “artificial virus” that can act as an immunogen, epitope-displaying nanocarrier, or delivery nanoplatform, is used to deliver vaccine antigens directly into a host cell [34]. Antigens can be conjugated via hydrophobic domains and lipid linkers on the surface of virosomes, additionally, integrating into the phospholipid bilayer, delivering into the cytosol of the antigen-presenting cells and stimulating humoral or cellular immunity [82]. The properties that virosomes share with viruses are based on their structure, where virosomes are safely modified viral envelopes that contain the phospholipid membrane and surface glycoproteins. As a vaccine delivery system, virosomes are biodegradable and biologically compatible with many host organisms [221]. Although liposomes, containing similar lipid bilayers to virosomes, have significant potential for delivering bioactive molecules both in vivo and in vitro, they cannot effectively deliver encapsulated molecules to the cells due to the low efficacy of fusing with cells [222]. By contrast, virosomes guarantee cellular delivery of epitopes due to viral envelop glycoproteins with receptor-binding and membrane fusion properties [222].

The virosome vaccine against influenza virus, named Inflexal V, is licenced by Europe, and Hepatitis A virus vaccine, named Epaxal, is approved in Asia, Europe, and South America [110]. There are significant advantages of the virosome delivery system, including high-quality and long-term antibody response, conformational stabilisation of antigen, the protection of antigen degradation, safety profile, and suitability to specific groups of people (e.g., the elderly, infants, or immunocompromised people) [110]. However, the significant disadvantage of virosome delivery systems can induce immune response due to viral glycoproteins on the surface, which can be eliminated via the use of hydrophobic polymers (such as polyethylene glycol and polyvinylpyrrolidone) to modify the surface of virosomes [82,222]. Moreover, rapid disintegration in the blood compartment is the second potential issue of virosome delivery systems [222]. To solve this issue, virosome conjugated with antigen is capable of reaching targeting sites within a short time after administration [222].

#### Clinical Development of Virus-like Particles and Virosomes Adjuvants

Influenza A virus (H3N2) was a large challenge in the quest for improved influenza vaccines due to the poor immunogenicity of the influenza vaccine in the elderly [81]. Shinde and co-workers used recombinant hemagglutinin trivalent nanoparticle vaccine (tNIV), which was produced on Sf9 insect cell-recombinant baculovirus platform containing wide-type virus sequences composed of conserved H3N2 epitope, as an influenza vaccine candidate, with the intramuscular administration into 330 adults (aged over 50 years old) (NCT03293498) [81]. This Phase 1/2 clinical trial found that this VLP-based influenza vaccine is safe and well-tolerated, stimulating a substantially higher level of neutralizing antibodies, with a long-protection immune response, compared to the approved Fluzone^®^ high-dose influenza vaccine [81].

A Phase 2 clinical study of a Chikungunya VLP vaccine (VRC-CHKVLP059-00-VP) was completed in infectious disease endemic areas in the Caribbean (NCT02562482) [80]. The vaccine was produced by human embryonic kidney (VRC293) cells transfected by DNA plasmid mainly with a single-stranded RNA encoding structural proteins E1 (function of cell fusion) and E2 (function of cell binding) and inactivated capsid DNA from the Chikungunya 37,997 virus strain [223]. Analysis of the 400 random volunteers within the trial proved the vaccine’s safety and tolerance and showed persistence of the immune response 72 weeks post vaccination [80]. Although the 16 mild-to-moderate spontaneous adverse events (related to the VLP) were more than that of the placebo vaccine group, all adverse events resolved, and the patients did not have clinical sequelae [80].

HPV VLPs self-assembled with the HPV L1 capsid protein to form high immunogenic particles following protein expression from yeast (or insect) cells [224]. A recently licensed HPV VLPs nonavalent HPV (9vHPV) vaccine, which triggers a broader immune response to prevent more subtypes of HPV than bivalent and quadrivalent vaccines, has shown significant efficacy and safety in clinical trials [225]. A 2014–2018 Phase 3 clinical trial confirmed the efficacy of the 9vHPV vaccine in 16- to 26-year-old males, with HPV quadrivalent vaccine (4vHPV) used as a positive control (NCT02114385) [79]. Following three doses of the 9vHPV (or 4vHPV) to two separate cohorts of people, both vaccines were shown to have a similar immunogenicity against four common HPV subtypes (6, 11, 16, and 18) [79]. However, the 9vHPV vaccine had significantly higher immune function than the HPV subtypes, 31, 33, 45, 52/, and 58 [79]. Although the two vaccines triggered similar ratios of systemic adverse events, six volunteers that received the 4vHPV vaccine had serious adverse events [79].

Embedding antigens in particles with support from flagellin adjuvants in VLPs could be a novel strategy for infectious disease vaccines. The free combination of embedded proteins may increase the immunogenicity of vaccines and the range of vaccine designs. The system’s remarkable success in HPV vaccine development offers a model for further vaccination studies and diseases. In fact, this is already occurring, as a novel VLP vaccine designed to target SARS-CoV-2 entered into Phase 1 clinical trials (NCT04818281) with appending early results [226].

Virosome delivery systems are involved in enhancing the immunogenicity of vaccines against infections. Phase 1 clinical trials of a HIV virosomal vaccine, which was intramuscularly, intradermally or subcutaneously administrated into healthy volunteers, showed a good safety record no adverse effects (NCT04553016) [82]. In this study, influenza-enveloped viruses are used to produce a virosome delivery system, combined with HIV-1 virulence antigens (including gp41 and p1 peptides), secreting cell-like helper T cells and macrophages [82]. Furthermore, an anti-malaria virosomal vaccine, named PEV3A, entered Phase 2 clinical trials using a virosomal formulation of two malaria peptide antigens, derived from the circumsporozoite protein and apical antigen-1 (AMA-1) of the K1 isolate of *P. falciparum* (NCT00408668) [83]. PEV3A is safe and well tolerated, induced high antibody titres and boosted the anti-malaria immune response compared to the placebo group [83].

Virus-like particle and virosome delivery systems are widely used against infectious diseases. Several vaccines adjuvanted with virus-like or virosome delivery systems (e.g., influenza vaccine, HBV vaccine, or HPV vaccine) are commercially available in the market and more clinical research of VLP and virosome adjuvants have entered in different phases, with the production of high antibody titres and induction of the adaptive immunity.

### 7.3. Synthetic Polynucleotides Adjuvants

The synthetic double-stranded RNA complex, polyribosinic: polyribocytidic acid (usually abbreviated Poly I:C or Poly(I:C)), activates innate and adaptive immune components. It mimics viral infections and elicits host immune responses by triggering specific pattern recognition receptors (PRRs) and TLR3 receptor [23]. The derivatives of Poly (I:C) are also effective adjuvants, including poly-IC12U and poly-ICLC. Poly-IC12U is less toxic than Poly I:C with a shortened half-life meaning it does not elicit antibodies to double-stranded nucleic acids DNA. Poly-ICLC retains the biological activity of the parent Poly(I:C) as well as significantly increasing resistance to nuclear solubilisation [23].

Synthetic oligodeoxynucleotides (ODNs) containing unmethylated cyclic di-GMP (CpG) motifs are similar in structural/function to bacterial DNA that bind to TLR9 [227]. Four classes of CpG ODN (type-D, -K, -C, -P) have been identified based on different structures and immune responses [86]. These four classes of CpG ODN contain a single motif in a central unmethylated ‘CG’ dinucleotide and flanking regions [86]. CpG ODNs, as an adjuvant, recognise TLR9 receptors (e.g., plasmacytoid dendritic cells and B cells) leading to the secretion of Th1 and pro-inflammatory cytokines and the induction of an innate immune response [227]. Further, CpG ODNs enhance professional antigen-presenting cells leading to induction of both humoral and cellular immune responses against infectious diseases [227]. With the commercial application of CpG as an adjuvant for a HBV vaccine, research into its potential into order vaccines are well underway [228].

#### Pre-Clinical and Clinical Development of Polynucleotide Adjuvants

A number of promising pre-clinical and clinical trials for polynucleotides adjuvants (e.g., Poly I:C, poly-ICLC and ODNs, and their derivatives) have been reported for infectious diseases, highlighting the promise for this adjutant class in the future of vaccine design. A few of these studies have been mentioned here and in Table 2.

Poly I:C combined with a recombinant HIV-1 envelope glycoprotein (gp120; mediates fusion of the viral envelope with the target cell membrane) was injected into mice demonstrating that immature dendritic cells presented antigens and produced increased levels of CD8^+^ cells with the strong cellular immune response against HIV when compared to the use of lipopolysaccharide, a TLR4 agonist adjuvant [229]. In addition to this, Poly I: C has also proven itself to be an effective mucosal adjuvant when used in combination with the HIN1 influenza hemagglutinin protein, PR8. Following intranasal immunisation into mice, this PR8/Poly (I:C) vaccine stimulated increased levels of IgA titres, with a potent humoral immune response in comparison to the influenza antigen alone [230]. In 2014, Saxena and co-workers physically combined plasma HIV-1 RNA with the TLR-3 agonist Poly-ICLC adjuvant as an HIV vaccine [85]. In this clinical study, the HIV vaccine was intramuscularly administrated to healthy volunteers and patients and provided the result that this vaccine produced no significant change in CD4^+^ T cell-associated HIV immunity following Poly-ICLC administration, despite safety and good tolerance (NCT0207195) [85]. Clinically, poly-ICLC has been indicated to be safe and enabled us to induce immunogenicity of vaccines, with the broad utilization of adjuvants against tumors rather than infectious diseases [231].

Rinatolimod (Ampligen^®^), a synthetic double-stranded RNA (Poly I: Poly C_12_U) combined with a formalin-inactivated whole virus antigen (NIBRG_14_) derived from a recombinant H1N5 avian virus entered Phase 1/2 clinical trials in 55 healthy volunteers (NCT01591473) [84]. Following intranasal immunisation, the influenza vaccine was tolerated, with the most common adverse effects being injection-site pain, fatigue and tolerated headache. This Rinatolimod (Ampligen^®^) adjuvant produced higher IgA titres and induced potent mucosal and humoral responses to against H5N1 with post-vaccine protection when compared to the un-adjuvanted groups [84]. Interestingly, increased mucosal and humoral immune responses occurred with the lower dosage levels of the vaccine, with further Phase 3 research to compare the immunogenicity of live-attenuated influenza vaccine adjuvanted with placebo, and low and high dose Rinatolimod underway (NCT00711295) [232].

Pre-clinical trials into the immunogenicity of CpG ODN-adjuvanted vaccines have involved various disease models [227]. For example, CpG ODNs have been extensively researched as adjuvants for HSV vaccines. Following intravaginal immunisation of mice with a HSV-2 vaccine adjuvanted with CpG ODN, rapid stimulation of a strong mucosal and protective Th1 immune response against lethal HSV-2 infection was observed [227]. Additionally, this HSV-1/CpG ODN-adjuvanted vaccine provided rabbits with significant Th1 immunity following generation of high neutralising IgA and IgG in both tears and sera [227]. Huang and colleagues conducted the oral administration of sonicated salmonella proteins combined with CpG ODN in newborn mice against salmonella enteritis infections, which showed higher IgG titres and stronger mucosal and systemic immune response, with higher survival than vaccination alone [227,233]. In addition to this, Klinman et al. demonstrated that intraperitoneal (or intranasal) immunisation of mice with a CpG ODN-adjuvanted licensed Anthrax Vaccine Adsorbed (AVA) induced a strong systemic response with significant protective immunity [234].

CpG 1018 (the only CpG adjuvant approved by the FDA for human vaccine use) is in a Phase 3 clinical trial (NCT02117934) for a hepatitis B vaccine (HBsAg-1018; HEPLISAV-B™) [235]. This Phase 3 clinical trial was conducted in 8374 patients, significantly reducing the number of hepatis B infections and associated morbidity and mortality in patients [235]. More recently, Kuo and co-workers selected a prefusion-stabilised SARS-CoV-2 spike protein (S-2P [SARS-CoV-2 S-2P]) as a novel vaccine antigen [236]. This COVID-19 antigen was combined with the FDA human-approved adjuvant (CpG 1018) and aluminum hydroxide, forming a COVID-19 vaccine candidate, which was intramuscularly delivered into mice and rats. The pre-clinical outcomes showed that this vaccine stimulated Th1 immune responses and produced high neutralising antibody titres with a strong protective immune response [236].

A Phase I clinical trial of the anthrax AV70909 vaccine candidate (AVA protein and CPG 7909 adjuvant [type of CpG ODN adjuvant that is specifically designed to agonise TLR9]) was conducted in 25 healthy adults, and resulted in increased antibody titres and the rapid induction of adaptive immunity compared with placebo groups (NCT01263691) [237]. In addition to this, Mullen et al. clinically assessed a malaria antigen Apical Membrane Antigen 1 (AMA1) antigen conjugated with this CpG 7909 adjuvant as an anti-malaria vaccine [87]. This Phase 1 clinical trial (NCT00344539) involved 75 malaria-naive volunteers and showed that the CPG 7909-containing vaccine induced higher anti-AMA1 IgG antibodies and increased levels of growth inhibition of homologous parasites with minor adverse effects (e.g., redness/pain at injection sites) [87]. Further, CpG 7909 forms part of the commercial influenza vaccine (Fluarix^®^), which has been injected into healthy adults as a Phase 1 clinical trial (NCT00559975) [238]. This trial demonstrated again supported the results seen in trials NCT01263691 and NCT00559975, with good disease protection and minor vaccine side effects [238].

IC31, a new two-component TLR9 agonist adjuvant, contains an 11-mer antibacterial peptide (KLKL (5) KLK) and a synthetic oligodeoxynucleotide (ODN1a) without the cytosine phosphate guanine (CpG) motifs [239]. This polynucleotide adjuvant has been applied to tuberculosis vaccine research, where the investigational TB fusion protein (H4:IC31(ASERAS-404)) derived from two *M. tuberculosis* (Mtb)-antigens (Ag85B and TB10.4) was intramuscularly administrated into 60 healthy volunteers [88]. The Phase 1/2 clinical trials (NCT02066428 and NCT02074956, respectively) resulted in the H4:IC31 (AERAS-404) vaccination being well tolerated with a good safety profile [88]. This H4:IC31 (AERAS-404) vaccine induced antigen-specific CD4^+^ T cell responses and produce cytokines (e.g., IFN-γ) following two-dose administration when compared to placebo groups [88].

### 7.4. Liposomes as Adjuvants (Mucoadhesive Antigen Delivery Systems)

**Figure 6:**
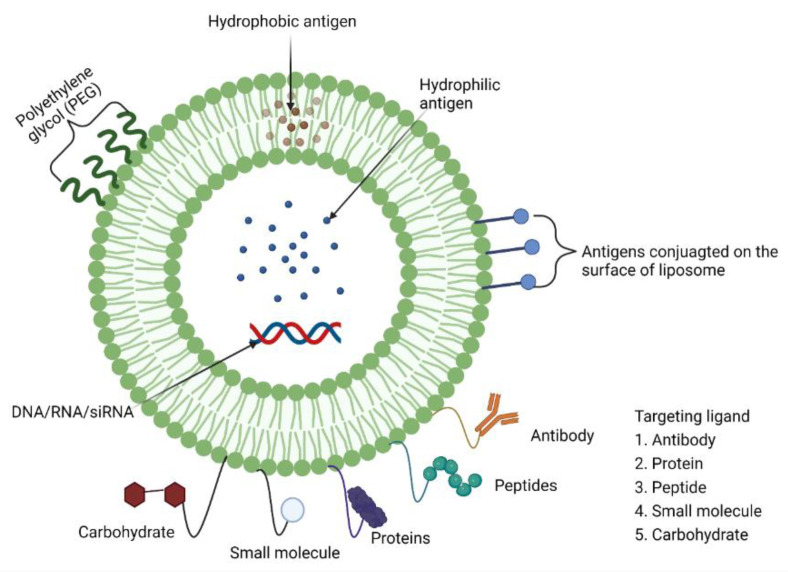
Liposome schematic.

Liposomes are small artificial vesicles of spherical shape that form an aqueous core entrapped by one (or more) lipid bilayers (Figure 6) [240]. The discovery of liposomes is traced back to 1956; however, the first application of liposomes as a vaccine delivery system was not until 1974 [241]. Liposomes are synthesised from natural (or synthetic) biodegradable, non-toxic and non-immunogenic phospholipids, which form an aqueous core [240,241]. Liposomes (including archaeosomes [liposomes made with one or more ether lipids that are unique to the domain of Archaeobacteria] and cationic and neural liposomes) are extensively used in vaccine applications due to their size, biocompatibility and physiochemical features [242]. Liposomes can be tailored (e.g., size, charge) to enhance vaccine efficacy, and this is achieved by using different lipids, targeting moieties (e.g., antibodies, proteins), synthesis/formulation procedures (to allow for size adjustment/refinement), and antigens (e.g., DNA, peptide) (Figure 6) [242].

In a liposome delivery system, water-soluble compounds (e.g., peptides, nucleic acids, and carbohydrates) are entrapped within the aqueous inner side of the liposome, while lipophilic compounds are located in the lipid bilayers [242]. Generally, depending on the nature of the antigen, they can be entrapped in a hydrophilic core, encapsulated in the hydrophobic bilayer, electrostatically bound to the liposomes surface, or absorbed via modification of acyl chains on the antigen [241]. Coincidentally, the liposome delivery system protects antigens from enzymatic degradation, increasing absorption rates and enhancing the bioavailability of vaccines [241]. Liposomes are used to target specific cellular sites, as well as release antigens to inflammatory sites, and as a result, have been used in many routes of vaccination (e.g., skin, oral, mucosal, and topical) [242]. Liposomes, combined with other types of adjuvants (including TLR agonists), increase the biostability of vaccine candidates, inducing a stronger humoral or cellular immune response [241]. Liposomes act as a delivery system for antigens and are effective immunostimulators, enhancing the exposure of antigens to the antigen-presenting cells and allowing for the maturation of CD4^+^ and CD8^+^ cells, and the stimulation of a potent humoral or cellular immune response [242]. However, the reproducible formulation of liposomes is a primary hurdle [241].

Lipid-based nanocarriers have been intensively used as vaccine adjuvants, with the ability to deliver nucleic acid antigens in vivo [243]. Lipid-based nanocarriers comprised of lipids and encapsulated nucleic acids (10–500 nm) have overcome system barriers, control the release time, and deliver antigens at the site of injection [243,244]. Formulations of lipid-based nanocarriers are affected by the lipid composition, the nucleic acid to lipid ratio, and formulation methods [243]. Cationic adjuvant formulation, CFA01, contains *N,N′*-dimethyl-*N,N′*-dioctadecylammonium (DDA) with the synthetic mycobacterial immunomodulator α,α′-trehalose 6,6′-dibeheneate (TDB) inserted into the lipid bilayers, as typical lipid-based nanocarriers [245]. Excitingly, CFA01 activates antigen-presenting cells, inducing B and T cell differentiation, with the capacity to present antigens to the relevant cell population [246]. CAF01 liposomes are highly polydisperse multivesicular vesicles, expressing an average particle size of about 450 nm [247]. CAF01 (or CFA01) is significantly cationic on the surface, with approximately +60 mv charge [247]. The lipid membrane of CAF01 liposome is in the solid ordered state at physiological temperature due to a phase transition temperature above 37 ℃. The use of a cationic liposomal formulation CAF01 has traced back to the 19th century and is currently involved in clinical research for vaccines against infectious diseases [247]. Ionisable lipids express a positive charge at low pH while remaining neutral at physiological pH [248]. The pH sensitivity of ionisable lipids is beneficial for the delivery of nucleic acid cargo for vaccines due to fewer interactions with the anionic membranes of blood cells in neutral lipids and enhancing the biocompatibility and stability of lipid nanoparticles [248].

#### Pre-Clinical and Clinical Development of Liposome Adjuvants (and/or Delivery Systems)

Liposomes, as a delivery system or adjuvant, have been widely involved in infectious disease vaccine development. Ghaffar et al. employed cationic liposomes to encapsulate a lipopeptide-based group A *Streptococcus* (GAS) antigen that was intranasally delivered into mice [249]. This GAS vaccine produced the higher IgA and IgG titres (detected after 5 months post immunisation) and strong mucosal and systemic immune responses when compared with the lipopeptide-based vaccine alone [249]. Polyethyleneimine (PEI)-adjuvanted cationic liposomes containing a GAS peptide antigen J8 were intranasally administrated into mice [250]. The outcome indicated that a high ratio of PEI induced a stronger immune response compared to the mice administrated a lower ratio of PEI [250]. The Pfs25 vaccine is preformed cobalt porphyrin–phospholipid liposomes, which encapsulate recombinant malaria protein antigen [251]. Immunisation of this Pfs25 vaccine was conducted in mice and rabbits, leading to spontaneous nano-liposome antigen particularisation and induction of high IgG antibodies humoral immunity [251].

Furthermore, this preformed cobalt porphyrin–phospholipid liposome co-administered with dual TLR ligands (GLA [TLR4 agonist] and 3M-052 [TLR7/8 agonist]) was assessed as a COVID-19 vaccine (SARS-CoV-2 protein antigen) by Abhyankar and colleagues [252]. Following intranasal delivery into SARS-CoV-2K18-hACE2 mice, the outcome of immunisation of this dual TLR ligand liposome system produced higher IgA titres and induced enhanced mucosal and systemic immunity compared with antigen alone [252]. COVID-19 mRNA-1273 is a lipid nanoparticle-encapsulated, nucleoside-modified messenger RNA (mRNA) strain that encodes the SARS-CoV-2 spike (S) glycoprotein as a COVID-19 vaccine candidate, which was intramuscularly administrated into 30,420 volunteers [89]. This Phase 3 clinical trial indicated that the vaccine is tolerated with no safety concerns, with 94% efficacy at preventing COVID-19 infection (including severe disease) (NCT04470427) [253]. BNT162b2, the Pfizer/BioNTech vaccine is a COVID-19 mRNA vaccine. Nucleoside-modified mRNA encoding the full-length spike protein of SARS-CoV-2 was encapsulated into ionizable lipid nanocarriers [248]. This BNT162b2 vaccine passed Phase 3 clinical trials (NCT 04368728) with 43,548 participants, which was safe and expressed 95% efficacy in preventing COVID-19 epidemics, with protective anti-COVID-19 immune response [248]. Smith and co-workers conducted the Phase 1 clinical trial of the cationic-lipid-based Vaxfectin^®^ adjuvant formulated with plasmid DNA vaccines encoding influenza virus H5 hemagglutinin (NCT00709800), as the influenza vaccine, intramuscularly administrated into approximately 100 healthy volunteers [254]. This clinical trial showed that Vaxfectin^®^-adjuvanted DNA plasmid influenza vaccine was tolerated well and stimulated the antibody immune response, with the protective immunity for pandemic control [254].

*Chlamydia trachomatis*, a tropical Gram-negative obligate intracellular bacterium, is responsible for bacterial sexually transmitted diseases worldwide, with over 90 million new cases of genital *C. trachomatis* infections occurring per year [255]. National screening programs and antibiotic drugs cannot decrease incidence of this disease [91]. Phase 1 clinical trials of a novel *C. trachomatis* recombinant protein subunit (CTH522) combined with the CAF01 liposomal adjuvant was conducted on healthy female volunteers aged between 19 and 45 by intramuscular administration (NCT02787109) [91]. No severe side effects were reported aside from minor adverse events at the local injection site [91]. This CTH522:CAF01 vaccine produced higher IgG titres and enhanced the mucosal antibody profile, with a more consistent cell-mediated immune response when compared with the alum-adjuvanted vaccine antigen (CTH522: alum) [91]. Furthermore, this CAF01 adjuvant combined with a recombinant fusion protein containing TB antigens (Ag85B and ESAT-6 (H1)) was assessed in Phase 1 clinical trials (NCT00922363) [91]. Following intramuscular immunisation into healthy human volunteers, no local or systemic side effects were observed [91]. A two-dose regime of this vaccine was shown to induce stronger antigen-specific T cell responses with post-vaccination protection after 150 weeks [91].

Liposomes, as a delivery system, encapsulate antigens and release them at the site of action [256]. To optimise liposome adjuvant immunology, liposome properties are engineered via the modification of size and charge, surface decoration with immune cell binding with antigens and the increase of in vivo circulation times [256]. Here, lipid-based nanocarriers (including cationic liposomes and ionizable lipids) that stimulate both cellular and humoral immune responses with high adjuvanticity have been used in human clinical studies against infections [256].

### 7.5. Polysaccharides as Adjuvants

Polysaccharide adjuvants (including chitosan, glucan, inulin, mannan, and some Chinese medical herbs) are a type of natural polymers containing glycosidically linked carbohydrate monomers [257]. Polysaccharides, as vaccine adjuvants, have been extensively involved in vaccine development due to their effective immunomodulating, biocompatibility, biodegradability, low toxicity, and safety profiles [257]. Polysaccharides are known to activate macrophages and NK cells, leading to cytokine and pro-inflammatory chemokines production and the stimulation of an innate immune response [257]. Furthermore, polysaccharides, as effective immunostimulators, induce humoral, cellular and mucosal immune responses to fight against infection [257]. Common polysaccharide adjuvants includes chitosan and its derivatives, such as glucan, inulin, and mannose.

Chitosan, derived from the exoskeleton of crustaceans, has a low toxicity, appropriate biocompatibility and biodegradability, supporting its extensive use in biomedical applications [257]. Chitosan is capable of producing pro-inflammatory chemokines, various cytokines (e.g., IL-1, IL-6, TNF), with the induction of both innate and adaptive immune responses [257]. In addition, chitosan derivatives (including chitosan quaternary ammonium salt and N,O-carboxymethyl chitosan) is known to activate systemic immune responses and mucosal immunity against infection [257].

Glucan adjuvants are comprised of repeating D-glucose units linked by glycosidic bonds, which are primarily derived from plants and microorganisms [258]. Glucans are classified into α- and β-glucans based on their conformations, where β-glucans are the primary glucan adjuvant used in vaccine applications as they bind to receptors (e.g., TLRs and dectin-1), stimulating both an innate and adaptive immune response.

Inulin, extracted from the roots of the flowering plant *Compositae*, contains a linear β-D-polyfructofuranosyl α-D glucose [258]. Inulin is divided into four different types (α, β, γ, and δ) where γ- and δ-inulin have been shown to induce cellular and humoral immunity [258].

Mannose, a sugar monomer of the aldohexose series of carbohydrates, is important in human metabolism (e.g., protein glycosylation). Mannose (as an adjuvant) binds macrophages and dendritic cells, stimulating innate immunity, and Th1 and/or Th2 immune responses [257].

#### Pre-Clinical and Clinical Development of Polysaccharide Adjuvants

From this adjuvant class, chitosan is the primary polysaccharide adjuvant researched in vaccine development against infectious diseases. McNeela et al. co-administered chitosan with cross-reacting material (CRM197) of diphtheria toxin, as a diphtheria vaccine that was intranasally delivered into mice [259]. This pre-clinical evaluation showed that the presence of chitosan elicited higher levels of IgG and IgA antibodies with robust Th2 immune responses [259]. Further, the intranasal immunisation of CRM197 diphtheria toxin plus chitosan elicited protective antibodies against the diphtheria toxin in guinea pigs, showing chitosan’s promise as a vaccine adjuvant [259].

An H5N1 influenza vaccine was adjuvanted with two chitosan derivatives (a glutamate salt from chitosan, CSN) and a trimethyl derivative of chitosan, TM-CSN. Following intranasal administration into a ferret model, both vaccines produced higher antibody titres with the strong Th2 and mucosal immune response and dramatically reduced replication in comparison to H5N1 influenza vaccine alone [260]. Interestingly, the TM-CSN induced higher protective antibodies than CSN in this study due to a high charge density of TMC to improve the paracellular permeability of mucosal epithelia [260,261].

A monovalent Norwalk virus VLP against acute gastroenteritis conjugated with MPL/chitosan (MPL/chitosan-VLP vaccine) was assessed in Phase 2 clinical trials (NCT00973284) [92]. Following intranasal immunisation, higher IgA antibodies and significantly less illness/infection was reported compared with placebo groups [92]. Advax, a delta inulin adjuvant, has been used in vaccination trials against the hepatitis B virus (HBV) with the induction of humoral and cellular immune response [93]. Phase I clinical trials of Advax combined with inactivated HBV (or yeast-expressed recombinant hepatitis B surface antigen; HBsAg) (ACTRN12607000598482) were promising when compared with the hepatitis B surface antigen alone [93]. Moreover, the Phase 1 clinical trial of a HIV-1 vaccine candidate co-administered with the Advax adjuvant was conducted in healthy adults and showed no vaccine-related severe adverse side effects (NCT00249106) [94].

Polysaccharides, as potential adjuvants, have been dispersed in the development of vaccines. However, the activity of polysaccharides and the appropriate process of producing and extracting polysaccharides remain largely unexplored [260]. The deep investigation of polysaccharide structure and biological function, the physicochemical properties and the modification of polysaccharide structure can enhance the humoral or cellular immunity, with a long-term protective immune response [260].

### 7.6. Polymeric Nanoparticle Adjuvants

**Figure 7:**
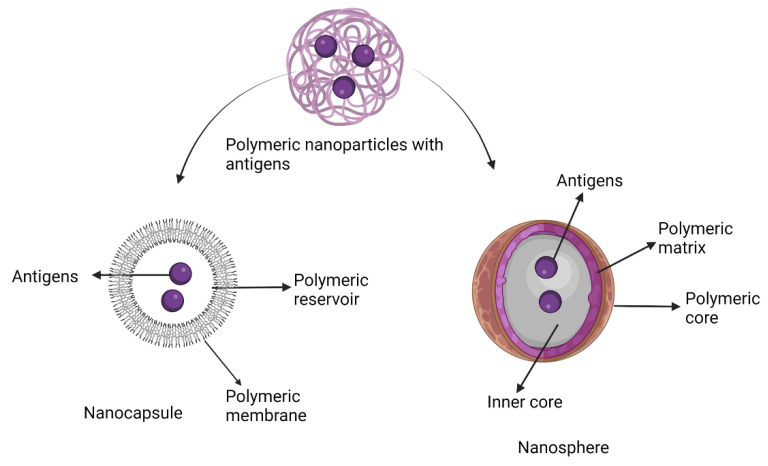
Schematic polymeric nanoparticles.

Polymeric nanoparticles, as carriers, have been tested in the development of vaccine adjuvants over recent years (Figure 7).

Nanoparticles contain both nanocapsules and nanospheres, with different morphology [262]. Nanocapsules contain an oily core from the vaccine candidates, which are surrounded by a polymeric shell, controlling the release profile of antigens from the core [262]. Nanospheres are a continuous polymeric network where antigens can be located inside or adsorbed onto the surface [262]. These two types of polymeric nanoparticles are defined as a reservoir system (nano capsule) and matrix system (nanosphere) [262].

Poly (lactic acid) (PLA) and poly (lactic-coglycolic acid) (PLGA) are artificial polymeric nanoparticles (encapsulating antigens and/or adjuvants). Both PLA and PLGA are the most common biodegradable and biocompatible polymer particles that have been licensed for human use (including structures and bone implants) and are approved by the FDA [263]. PLA/PLGA particles prolong antigen release and reduce the number of repeated doses required for long-term protection, leading to a reduction in vaccination costs [263]. PLA and PLGA particles have been used as a vaccine adjuvant to improve the efficacy of antigen-presenting cell uptake and MHC-I and MHC-II processing, leading to antigen presentation toward Th1 or Th2 immune responses [263]. Although PLA/PLGA-based micro-particles have been used as vaccine adjuvant/delivery systems, inappropriate conditions of encapsulating methods (organic solvent/water interfaces, shear or cavitation forces, freezing and drying) significantly degrade vaccine antigens [263]. These problems are being addressed through optimised manufacturing methods or combination with stabilising agents (e.g., Mg (OH)_2_ other proteins, surfactants or sugars) [263].

#### Pre-Clinical and Clinical Development of Polymeric Nanoparticle Adjuvants

Polymeric nanoparticles express significant immunological properties, recognition of antigen-presenting cells, cytokine stimulation, and are immunostimulatory for the vaccine candidate [264]. Polymeric nanoparticles make promising delivery systems and adjuvants for a range of vaccine antigens, where their popularity stems from their ease of synthesis, biocompatibility, their biodegradable nature, and the fact they are non-immunogenic, non-toxic and fairly inexpensive [220]. Disadvantages of polymeric nanoparticles include the stability of antigens in microcapsules and optimal dose problem [264]. Numerous different polymeric nanoparticles exist, with the most frequently encountered types comprised of chitosan, PLGA and PLA [220].

PLA co-administrated with HIV p24 protein was intravenously administrated into mice [95]. This pre-clinical assessment showed that encapsulation of the HIV p24 antigen with PLA induced a higher IgA titre and a stronger Th1 immune response against HIV infection compared with HIV p24 antigen alone. DNA-based HBV antigen was entrapped with PLGA adjuvant as an HBV vaccine, which was delivered into the murine model [265]. The immunisation of PLGA adjuvant produced higher antibody titres with a strong humoral immune response in comparison to the HBV vaccine alone [265].

DermaVir, an experimental HIV/AIDS topical vaccine consists of an HIV-1 antigen encoding DNA plasmid encapsulated into the polymeric adjuvant in nanoparticle size [266]. DermaVir was transdermally administrated into 36 HIV patients under a patch after skin preparation [266,267]. The Phase 2 clinical trial finished in 2020 and the results indicated that DermaVir vaccine is safe and tolerable, with a long-lasting HIV-specific immune response compared to placebo groups (NCT00711230) [267].

Polymeric nanoparticles, a common delivery system, are adjuvanted with pathogenic antigens due to the controlled release of antigens and simple and cost-effective production, while optimal dose problems can be eliminated via appropriate delivery routes and the modification of nanoparticles. Although polymeric nanoparticles have been tested in pre-clinical trials or clinical studies of vaccines against infections, this nanoparticle is widely used in anti-tumor therapeutic vaccines or drugs.

### 7.7. Glycosphingolipids as Adjuvants

Glycosphingolipids (GSL) are a class of lipids (specifically, sphingolipids) and make up part of the cell membrane. GSL consist of a hydrophobic ceramide and a glycosidically bound carbohydrate part and exhibit immunogenicity in immunised animals [268]. Recent studies found α-linked monosaccharyl ceramides (derived from marine sponges and bacteria) bind to natural killer T (NKT) cells, as iNK agonists, and elicit the secretion of cytokines [268].

NKT cells, as a bridge between innate and adaptive immunity, are a small and unconventional T cell subset [269]. Major NKT cells form an invariant T cell receptor (TCR) α chain rearrangement-Vα14Jα18 in mice but V-α14Jα18 in humans, with limited Vβ chains diversity, called invariant NKT (iNKT) cells [269]. Unlike conventional T lymphatic cells, which bind to peptide antigens in the context of MHC class I and class II molecules, iNKT cells bind to lipid (or glycolipid) antigens secreted by the MHC class I-related glycoprotein CD1d, which rapidly secretes cytokines, mediating cytotoxicity [269,270]. Secreted cytokines induce the activation of NK cells, macrophages, and in combination with CD40/CD40L interactions form mature dendritic cells, which affect both innate and adaptive immunity [269]. Thus, the design of an appropriate iNKT cell agonist, as the adjuvant, used in vaccines, is able to improve the efficacy of vaccines by augmenting the magnitude of humoral and cellular immune response [269]. Currently, iNKT cell agonists (and analogues) as vaccine adjuvants have been triggered to infectious diseases and cancers.

**Figure 8:**
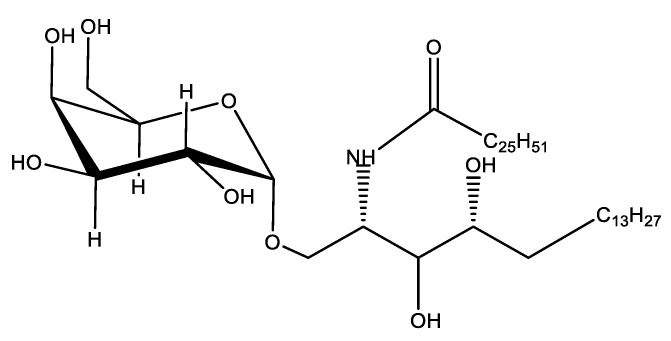
Structure of α-Galactosylceramide.

The glycoplid, α-Galactosylceramide (α-GalCer; Figure 8), a natural product of mollusks, activates iNKT cells to trigger to endogenous glycolipids on tumor cells and eradicate them, simulating adjuvant properties for adaptive T cell immune responses [271].

#### Pre-Clinical and Clinical Development of Glycosphingolipids Adjuvants

Tsujii and co-workers demonstrated that inclusion of α-GalCer (Figure 8) in a malaria vaccine significantly improved protective immunity within two weeks, when compared to the vaccine alone through the activation of iNKT cells leading to secretion of Th1 type cytokines (INF-γ) and CD8^+^ T cells against the malaria parasite [269]. In addition, α-GalCer has been assessed in the development of HIV vaccines, with current studies focusing on a combination DNA vaccine with α-GalCer (pADVAX) [269]. This pADVAX vaccine stimulated CD4^+^ and CD8^+^ epitope-specific IFN-γ responses and high IgG responses in mice with a lower immunogenic dose when compared with the DNA-only vaccine [269]. This reduced vaccine dose was consistent with influenza research by Ko’s group, which found that nasal co-administration of α-GalCer with an influenza virus vaccine enhanced strong humoral, cytokine and T cell responses, and required a lower dose than the antigen alone [269]. In 2007, Youn et al. concluded that the specific humoral immune responses were enhanced via administration of influenza vaccine with α-GalCer as an adjuvant [269]. Furthermore, immune cell-mediated responses were stronger than with vaccine alone based on the release of various Th2 cytokines (e.g., IL-4 and IL-5) [269]. For influenza vaccines, both Th1 and Th2 cytokine environments are significant, and are enhanced via α-GalCer addition [269]. In 2017, a DNA influenza vaccine, encoding influenza A virus matrix protein 2 (M2) and adjuvanted with α-GalCer was intramuscularly immunised into mice leading to a higher IgG titre and Ig2a/IgG1 ratio with long-term protective immunity compared with the DNA alone vaccine [272]. Although α-GalCer has been used in pre-clinical investigations for influenza vaccines, currently no clinical trials of influenza vaccines combined with α-GalCer adjuvant are reported.

Woltman and co-workers combined an HBV protein antigen with synthetic α-GalCer (KRN 7000) and following intravenous administration to participants, the Phase 1/2 clinical trial showed evidence of safety despite the α-GalCer adjuvant being poorly tolerated and now a suitable adjuvant-vaccine against HBV (NCT00363155) [96].

Overall, α-GalCer as an adjuvant induced potent humoral and cellular immune responses, where pre-clinical studies of influenza vaccines adjuvanted with α-GalCer have been tested in animal models without transition to clinical trials. α-GalCer has huge potential in the development of therapeutic vaccines against cancers (e.g., lung cancer, breast cancer, and leukemia) with significant progress made in both pre-clinical and clinical studies.

## 8. Tensoactive Adjuvants

Saponin adjuvants, discovered in 1925, were originally sourced from crude extracts of bark or plant skins [273]. However, the development of saponins accelerated in the 1950s after they were used as a veterinary vaccine adjuvant against foot and mouth disease virus [274].

**Figure 9:**
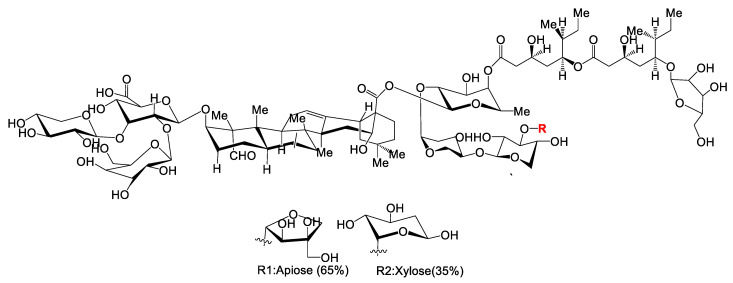
QS-21 adjuvant structure.

It was found that natural glycosides of steroids, steroid alkaloids and triterpene compounds found in saponins altered cellular membrane-stimulating cells to produce signalling factors, resulting in an increased immune response [275]. Quil-A, an extract of *Quillaja sapomari*, and its derivatives are the most extensively used saponins for adjuvant purposes [273]. As Quil-A (a heterogeneous mixture) is too toxic for human use, isolation and analysis of individual fractions (e.g., QS-21; Figure 9) from Quil-A are the most frequently used saponins for adjuvant purposes in infectious disease vaccines (e.g., malaria, hepatitis; Table 2) [273].

Saponin-type adjuvants (e.g., QS-21) effectively generate Th1 cytokines, cytophilic antibodies and strong antigen-specific cytotoxic T cell responses to induce humoral and cellular responses [273,276]. This is presumably due to the surface lectins of antigen-presenting cells interacting with the carbohydrate region of QS-21, facilitating antigen uptake and resulting in enhanced T and B cell responses [276]. Balanced IgG1 (promoted via Th2 immunity) and IgG2a (enhanced by Th1 immunity) production has also been observed in a pre-clinical study using inactivated Aujeszky’s disease virus as the antigen with QS-21 in mice [277]. It has alternatively been proposed that QS-21 and the antigen are absorbed through endocytosis, since QS-21 was found to have high affinity to endosomal membrane cholesterol on the dendritic cell [276]. Absorption through endocytosis resulted in the stimulation of CD8^+^ T cells and cytotoxic lymphocytes [276]. Despite the significant success of QS-21 as a vaccine-adjuvant in the last few decades, it is necessary to deeply understand its mode of action, accelerating the development of new vaccines [276]. In addition, the Matrix-M adjuvant, a new saponin analogue extracted from Q. saponins and formulated with cholesterol and phospholipids into nanoparticles [278]. The Matrix-M adjuvant boosts Th1 and Th2 responses, improves immune cell trafficking and allows for antigen dose-sparing, and as a result, has been widely used in pre-clinical and clinical vaccine trials due to its safety and efficacy [278].

While saponin adjuvants show great promise in vaccine development due to their ability to produce a desired immune response, continuously obtaining these compounds on a commercial scale from natural sources is unsustainable and the technology needed to synthetically manufacture them in a reproducible manner is lacking [279]. It has also been noted that the stability of these adjuvants is low, with reports of dosage-related toxicity leading to adverse effects (e.g., hemolysis) [279].

**Figure 10:**
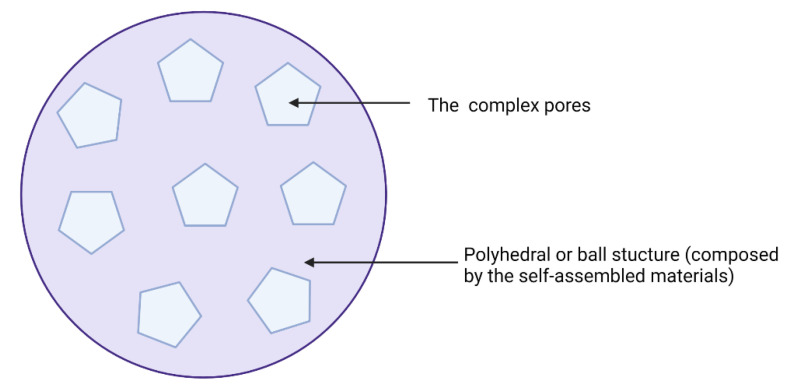
Schematic of immune-stimulating complexes (ISCOMs).

Immune-stimulating complexes (ISCOMs) are spherical, open cage-like structures (40–60 nm in size, Figure 10) that spontaneously form when cholesterol, phospholipids and Quil-A saponins (e.g., QS-21 or its variants) are combined in a specific stoichiometry via self-assembly [280]. Following assembly, a colloidal dispersion is produced using different methods (e.g., ultracentrifugation, dialysis, lipid film hydration, ethanol injection) [280]. As the main immunostimulator in the adjuvant, Quil-A enhances the secretion of antibodies and cellular immune responses [281]. Cholesterol serves to combine Quil-A with the phospholipids, which form the cage-like geometry and improves the affinity of the adjuvant with cellular membranes, and is proposed to improve the endocytosis of antigens by antigen-presenting cells [281]. The antigens are encapsulated in the complex pores (or are bound on the complex surface) through hydrophobic (or electrostatic) interactions to form a unique vaccine delivery system that is antigen-specific [281]. Notably, while the adjuvant capacity is retained, the toxic haemolytic activity of Quil-A saponins is significantly reduced when these adjuvants are combined with cholesterol [281].

### Pre-Clinical and Clinical Development of Saponin and Its Derivatives

QS-21 is an effective immune-stimulant, but limitations include the potential for dose-related toxicity, low chemical stability, low extraction yields (from natural sources), and an incomplete understanding of the mechanism of action [282]. To overcome these limitations, structural modifications of QS-21 adjuvants through chemical synthesis have been undertaken over the last decades, with nearly 50 saponin analogues discovered [281]. The derivatives with only minor structural changes were tested with a range of vaccine antigens in vivo to determine their immunogenicity and safety [282]. During pre-clinical studies with the QS-21 variants, the structure-activity relationship of QS-21 was analysed, as well as the immunostimulatory potential of the different regions of the molecule [276]. It was discovered that changing the linear oligosaccharide domain via simplification of the sugar acyl chain created more effective QS-21 variants [282]. For example, the echinocystic acid variant 74 (SQS-1-8-5-18), which has low toxicity, high stability and high adjuvant immunogenicity, was discovered through this structure-activity relationship method.

Wang et al. successfully synthesised unnatural analogues of QS-21 [279]. These unnatural saponins contained an aliphatic chain connected to the C3 trisaccharide domain through a stable amide bond (rather than an acyl side chain as in QS-21) [279]. These QS-21 analogues were more effective at potentiating a serum antigen-specific IgG response during the adaptive immune response than QS-21 [279]. Marciani et al. developed the semi-synthetic saponin analogue GPI-0100 [280]. This synthetic compound completely removed the original acyl side chain and subsequently incorporate a dodeculamime chain via amide formation [279]. GPI-0100 elicited both a humoral and cellular immune response, with less toxicity compared to QS-21 [279].

Matrix-M adjuvant combined with the full-length SARS-CoV-2 spike (S) glycoprotein was developed as a COVID-19 vaccine (NVX-CoV2373), where Phase 1/2 clinical trials of NVX-CoV2373 were completed (NCT04368988) [97]. Phase 3 clinical trials of this vaccine were conducted in Europe (EudraCT number, 2020-004123-16), demonstrating that a two-dose vaccination of NVX-CoV2373 vaccine was safe and 89.7% effective against symptomatic COVID-19 [98].

The vaccine, H5N1, for the pathogenic avian influenza virus, failed to provide a satisfactory mucosal immune response and completely stop the spread of the disease after its significant outbreak in 2003 [283]. The antigen in the intranasally administered vaccine (AIV-H5N1-inactivated vaccine) was produced by the strain of A/Chicken/Denpasar/01/2004(H5N1) in eggs and inactivated by formaldehyde [283].

ISCOMS-AbISCO-300 (a subtype of ISCOMs) and inmunair (INM: the mixture of inactive Propionibacterium acne with lipopolysaccharide used in animal vaccines) were incorporated as adjuvants with the same AIV-H5N1-inactivated antigen for enhancement of immunogenicity following intranasal immunisation [283]. In a pre-clinical trial, chickens and mice were vaccinated with INM co-administered with the antigen, ISCOMs with the antigen, ISCOMs combined with INM and the antigen, and the antigen alone [283]. The combination of INM and ISCOMs increased the immune response of mice and chickens, while administration of INM alone did not enhance the immune response of mice [283]. ISCOMATRIX (derived from the ISCOM) has been widely used in hepatitis C virus and influenza in humans [149]. Phase I clinical trials of H7N9 influenza virus combined with ISCOMATRIX showed that addition of the ISCOMATRIX adjuvant enhanced higher antibody immune response against avian influenza than the H7N9 vaccine alone (NCT01897701) [99]. ISCOMATRIX, combined with other TLR agonist adjuvants (e.g., CpG ODN, MPL), as therapeutic or prophylactic vaccines, has also been investigated [284]. Overall, the immunogenic function of ISCOMs derivates and their strong potential in combination with other adjuvants offers new possibilities for novel adjuvants in the future [284].

## 9. Protease Adjuvants

Proteases are divided into different families (e.g., cysteine, serine, aspartic, and threonine proteases) and have significant roles in protein proteolysis [285]. Papain-like cysteine proteases (considered the most abundant type of cysteine proteases) are derived from virus, bacteria, yeast, protozoa, plants and animals, and contain a nucleophilic cysteine thiol at the active site [285]. This protease class stimulates immune responses towards a Th2 immunity and is a potential adjuvant in vaccine development [12].

### Pre-Clinical Development of Protease Adjuvants

Papain-like cysteine proteases have been used in vaccine development against schistosomiasis [12]. *Schistosoma mansoni* glyceraldehyde 3-phosphate dehydrogenase (SG3PDH), peroxiredoxin (TPX), and other larval excretory–secretory products (ESP) are *Schistosoma* antigens that induce Th1 and Th17 immune responses with a non-protective natural infection [100]. Ribi and colleagues selected recombinant SG3PDH and TPX-derived peptides combined with papain which were injected into a murine model [100]. This pre-clinical trial demonstrated that the immunization of cysteine proteases produced the higher antibody titres and boosted immune responses toward a Th2 type [100].

Cysteine and serine proteases, as an adjuvant, produce a high level IgE antibodies and induce a Th2 immune response where these adjuvants have been widely used against allergic reactions and asthma (rather than infections) [286].

## 10. Combination Adjuvant Systems

Adjuvant systems (AS; developed by GlaxoSmithKline) are combinations of various classic adjuvants and immune stimulators to stimulate the adaptive immune response against pathogens [287]. Immune stimulants (alum adjuvants, TLR agonists or QS-21) have been incorporated into adjuvant systems with oil-in-water emulsions or liposomes [110]. These components synergise with one another to stimulate a broad range of immune responses, and the selected groupings of adjuvants have shown superior safety and immunogenicity [110]. Currently, various adjuvant systems (including AS01, AS02, AS03, and AS04) have been widely developed and a few have been conducted in the clinical trials, with AS01 (e.g., Malaria vaccine RTS, S or Mosquirix; Herpes zoster vaccine HZ/su or Shingrix), AS03 (pre-pandemic H5N1 vaccine and Pandemic H1N1 influenza vaccine) and AS04 (HPV vaccine Cervarix; HBV vaccine Fendrix) used in commercial vaccines [287].

The combined saponin-liposome adjuvating system (AS01) uses MPL as a TLR4 agonist with the inclusion of the saponin QS-21 [288]. This system has enhanced CD8^+^ cell-mediated immunity and cellular responses. Here, MPL directly promotes antigen-presenting cell expressing TLR4, activating the secretion of cytokines and co-stimulatory molecules [288]. QS-21 stimulates antigen-specific antibody responses and cytotoxic T cell immune responses [288]. AS01 is required to achieve the highest antigen-specific adaptive response [288]. QS-21 directly activates the antibody immune response, which is significantly enhanced by the addition of MPL [288].

The AS02 adjuvant combines a water-in-oil emulsion with MPL and QS-21, and has been shown to provide dominant cellular responses [289]. This synergy between QS-21 and MPL produces high levels of IFN-γ, a typical cytokine for CD4-type cellular response, where this water-in-oil emulsion stimulates both humoral and cellular immune responses [289]. Both AS01 and AS02 adjuvants were first developed for the zoster vaccine against malaria and herpes where they entered clinical trials (Table 2) [289].

**Figure 11:**
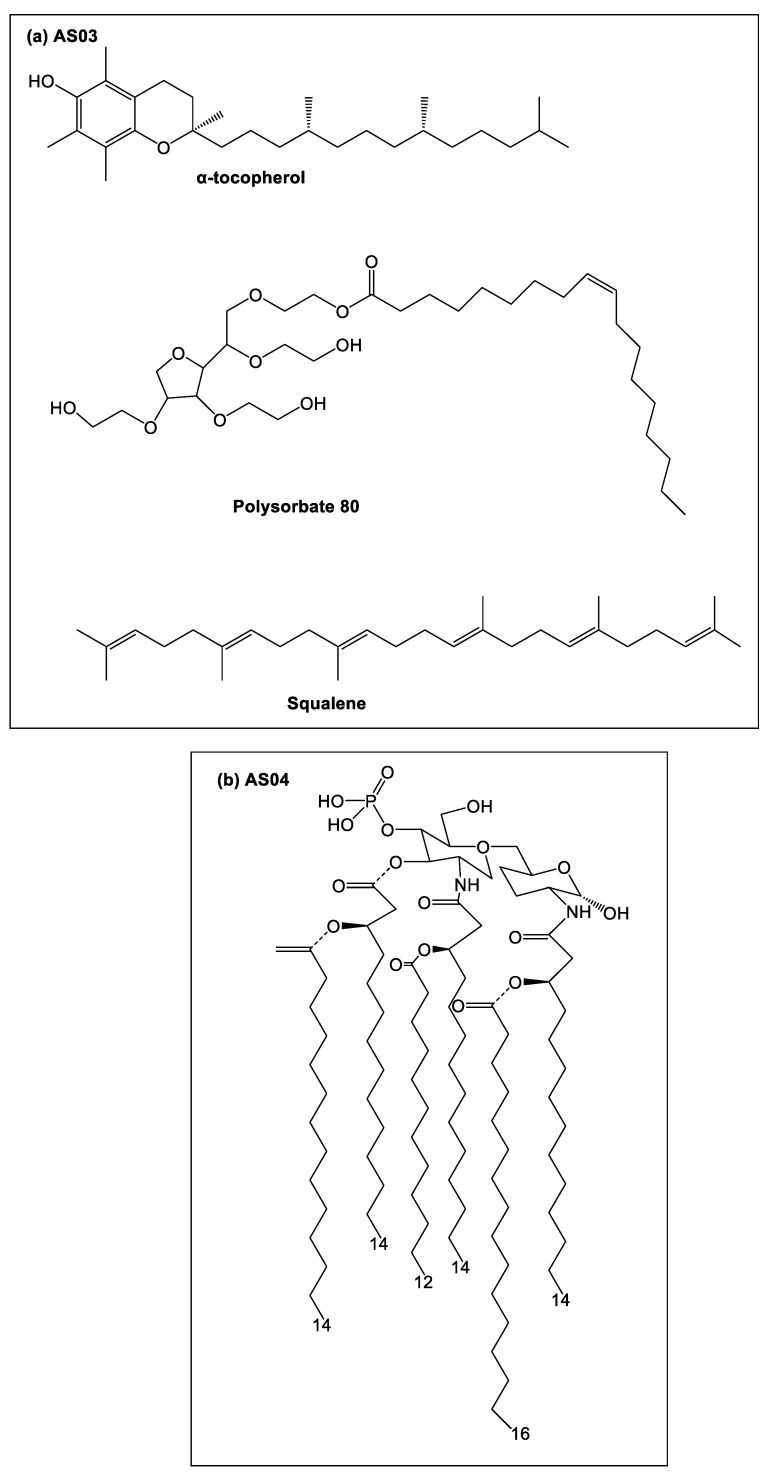
(**a**) Components of adjuvant system AS03 (assembled from squalene, polysorbate 80 and α-tocopherola [a type of vitamin E]). (**b**) MPL structure of adjuvant system AS04 as a TLR4 agonist which is mixed with alum.

The AS03 adjuvant system is comprised of a squalene-based oil-in-water emulsion, with the addition of DL-α-tocopherol (antioxidant) and tween 80 (polysorbate 80, as surfactant) (Figure 11). AS03 has a similar mechanism to that of MF59, with the stimulation of both Th1/Th2 cells leading to humoral and cellular immune responses [34]. In addition, AS03 also produces chemokines and pro-inflammatory cytokines, which allow for the recruitment of immune cells (e.g., macrophages), improving the secretion of antibodies [34].

AS04 (Table 2) is a combination alum-based adjuvant system where alum is physically mixed with the TLR4 agonist, monophosphoryl lipid A (MPL) as a licensed adjuvant for human use [290]. AS04 has been used in the development of the HPV vaccine, Cervarix^(R)^. This adjuvant system retains the core adjuvating properties of alum, while also promoting a Th1 immune response associated with the TLR4 agonist, resulting in a more effective adjuvant. MPL (in the AS04 adjuvant) retains its capability to bind to TLR4 and stimulate an innate immune response, activating NF-κB and secreting various cytokines and chemokines at the injection site [287]. Pro-inflammatory chemokines and cytokines promote the secretion of recruited immune cells (e.g., monocytes, macrophages) to form mature antigen-presenting cells, which migrate to the lymph nodes activating both Th1 and Th2 immune responses [287]. AS04 overcomes the limitations of alum salts, which only stimulates Th2 immune responses, and the number of pro-inflammatory cytokines which are neither enhanced nor inhibited by alum salts [287]. Lastly, combined with alum, the local cytokine responses stimulated by MPL are significantly prolonged at the injection site [287].

### Pre-Clinical and Clinical Development of Combination Adjuvant Systems

Over the long-term co-evolution of *P. falciparum* with its human host, *P. falciparum* has developed multiple methods of escaping the impacts of human immune responses, leading to increased difficulty in malaria vaccine development [102]. One of the most significant challenges has been polymorphic protein expression on the parasite’s surface [102]. The FMP2.1/AS02A vaccine candidate contains one of the conserved *P. falciparum* polymorphic proteins derived from the apical membrane antigen 1 (AMA1) co-administered with AS02 adjuvant [102]. A Phase 2 clinical study of the FMP2.1/AS02A vaccine was completed in 2018 in Mali with 400 children (1 to 6 years old) who were identified as having a high risk of infection to malaria compared to their adult counterparts (NCT00460525) [102]. A rabies vaccine from Chiron Vaccines, which does not express specific antibodies against *P. falciparum*, was used as a control [102]. The outcomes showed that children vaccinated with FMP2.1/AS02A induced a CD4^+^ T cell response [102]. It is possible that the AS02 adjuvant affected the phenotype of AMA1-specific CD4^+^ T cell response due to the induction of T cell response towards to Th1 (or Th2) responses [102]. The adjuvanted vaccine was sufficient to prevent most types of *P. falciparum* infection [102].

The Walter Reed Army Institute of Research (WRAIR) and GSK developed the RTS, S malarial vaccine in 1987 [291]. The ‘R’ in RTS, S vaccine represents the central repeat region, made up of the chain of N-acylneuraminate-9-phosphate (NANP) amino acid tandem repeat tetrapeptides. ‘T’ represents the immune dominant segregated epitopes of T lymphocytes, and ‘S’ is the surface antigen of Hepatitis B [291]. This RTS, S malaria vaccine contains the circumsporozoite protein and an adjuvant system (AS01 and AS02) and was clinically assessed in children and infants [148]. The AS02 adjuvant system contains an oil-in-water emulsion with MPL and QS-21 [292]. Compared to AS03 (an oil-in-water emulsion) and AS04 (a combination of aluminum and MPL) adjuvants, the recombinant protein malaria antigen with AS02 induced higher antibody levels and a protective immune response against malaria [292]. Despite the clinical results, RTS, S/AS02, AS01 (Mosquirix™) as a new combination adjuvant system which included liposome, QS-21, and MPL, was included in malaria vaccines due to the induction of antigen-specific cytotoxic T-lymphocyte response and strong humoral immunity [292].

In 2009, Polhemus et al. conducted Phase 2 clinical trials of RTS, S/AS02A and RTS, S/AS01B in adults in high malaria-transmission areas with the significant illustration of stronger immunogenicity with RTS, S/AS01B compared with RST,S/AS02A (NCT 00197054) [293]. Thus, development of RTS, S/AS02 was stopped and the RTS, S/AS01 combination adjuvant systems were advanced to Phase 3 clinical trials in Africa (NCT00866619) [101]. Here, the RTS, S/AS01 malaria vaccine reduced malarial morbidity and severe malaria by approximately half 12 months after injection in infants (aged between 5 to 7 months), while the mortality of malaria did not significantly decrease post vaccination [101].

RTS, S/AS01 combined with a plasmodium subunit antigen was shown to secrete antigen-specific immunoglobulins and CD4^+^ cells, with the stimulation of both cell-mediated and antibody immune responses, effectively blocking sporozoite colonisation of hepatocytes and protecting against new, *P. falciparum* [291]. However, Phase 3 clinical trials of this RTS, S/AS01 malaria vaccine did not provide sufficient protection due to passage of time [291]. Further, potential safety risks occurred in the young-testing group, [291] meaning further optimisation of the RTS, S/AS01 malaria vaccine to focus on the prolonged immunogenicity and elimination of the safety problems in children is necessary [291].

GSK Biologicals’ licensed HPV vaccine (GSK 580299) has proven safety, immunogenicity and efficacy following the completion of Phase 1, 2 and 3 clinical studies [294,295,296]. The vaccine consists of HPV-16 and HPV-18 L1 protein VLPs formulated with the AS04 adjuvant system of MPL adsorbed on aluminum hydroxide salt [294,296]. However, to confirm the efficacy of the vaccine in women living with HIV (as this demographic has a higher risk of being infected by HPV), a Phase 4 clinical trial was completed in 2019 (NCT01031069) [297]. In this trial, the AS04-HPV-16/18 vaccine was compared with the licensed 4vHPV vaccine in a total of 546 volunteers, where half of the individuals were women living with HIV [297]. The data from this trial indicated that the AS04-HPV-16/18 vaccine induced higher immunogenicity than the 4vHPV vaccine [297]. The positive result also confirmed the important role of adjuvants and offered an effective method for using adjuvants to combine target antigens for improved immunogenicity [297].

The AS03 adjuvant combined with soluble perfusion-stabilised spike trimers (preS dTM) from SARS-CoV-2, as a new COVID-19 vaccine candidate was administered as two doses to non-human primates [103]. The result of this pre-clinical assessment showed that the AS03-adjuvanted vaccines elicited a strong antibody immune response and protected the upper and lower airways against COVID infection [103]. Further, the large secretion of IgG antibodies offered protection from SARS-CoV-2 infection [103]. In addition, the SARS-CoV-2 recombinant protein nanoparticle vaccine (GBP510) adjuvanted with AS03 was intramuscularly administered into 328 participants aged from 15 to 85 years of age [104]. This Phase 1/2 clinical trial (NCT04750343) proved that vaccination was safe and well tolerated, with minimal adverse side effects. The AS03-adjuvanted vaccine produced higher IgG titres and enhanced a stronger Th2 immune response with post-vaccine protection response following two vaccine doses when compared to the un-adjuvanted vaccine [104].

To compare the adjuvating potential of MF59^®^ and AS03, a study was conducted. The research considered the pdm09 vaccine (for H1N1), where both adjuvants were tested in clinical trials (NCT00616928) [105]. This study was conducted in adults and children, with separated control (pdm09 vaccine without adjuvants) and test groups (pdm09 vaccine with adjuvants). An indirect comparison meta-analysis of randomised controlled test was used to compare the immunogenicity and safety of MF59^®^-adjuvanted, AS03-adjuvanted and un-adjuvanted pdm09 vaccine in different aged groups (including adults and children) [105]. In terms of safety, both adjuvants caused higher risk of erythema and pain at the injection site compared to the un-adjuvanted subjects [105]. AS03 also had a higher result of fatigue and injection site pain versus MF59^®^ in adult test groups [105]. No significant differences in safety were recorded among children [105]. Adjuvant groups showed higher immune responses than their respective control groups, with AS03 proving favourable [105]. In cases, though, MF59^®^ appeared to be more effective in generating an immunological response [105]. Therefore, AS03 is an effective adjuvant in H1N1 influenza vaccine development.

Adjuvant systems (AS0x) are designed to induce adaptive immunity against pathogens in specific aged groups (e.g., children, the elderly, and immunocompromised populations) [287]. AS03 and AS04 have been approved by the United States and European drug organisation, with wide use as part of pandemic influenza vaccines and prophylactic vaccines against HPV or hepatitis B virus [287]. AS02-adjuvanted vaccines have been tested against malaria, hepatitis B or in conjunction with cancer immune therapy [287].

## 11. Perspective and Conclusions

As effective immune stimulators, adjuvants dramatically change the safety and efficacy of a vaccine antigen. Hence, adjuvant development is an important variable in vaccine development, where adjuvant selection should be considered with a comprehensive understanding of the immune system and antigen-triggered responses. To fully understand adjuvant capacity and define stimulated immune responses, studies on adjuvant mechanisms are essential. Classical adjuvants, including alum, MF59, AS03, or AS04, are commercial adjuvants that have a broad use in vaccine development.

On the other hand, adjuvant delivery systems such as virus-like particles, virosomes, liposomes, and polymeric nanoparticles, have the potential to act as potent immune adjuvants. These systems control the release of antigens to specific sites, protect antigen bioactivity, and enhance antigen circulation times, stimulating a potent and protective adaptive immune response. However, the stability and toxicity of these delivery systems and optimal dose problems of vaccines are significant disadvantages, overcome through tailored engineering using targeted functional groups.

Cytokines, chemokines, polysaccharides, and glycosphingolipids are known to induce a humoral immune response, as well as a cellular immune response against infections, while also widely being used in anti-tumour vaccines with the induction of potent cellular immunity observed through several clinical studies.

The discovery of adjuvant derivatives improves adjuvant selection from the possible emergence of high-quality adjuvants. For example, the novel synthetic process of QS-21 is an outstanding discovery for QS-21 derivatives; increasing the number of QS-21-derivatives with enhanced adjuvating properties and reduced side effects is an important step in adjuvant design for infectious disease vaccines. In addition, microbial or bacterial adjuvants (e.g., flagellin, lipopolysaccharide, cholera toxin, and bacillus Calmette–Guérin) are selected and inactivated from pathogenic bacteria, stimulating the adaptive immune response against infections, with probable unexcepted side effects. Although the single use of adjuvants stimulates strong adaptive immune responses, the combination of more than two adjuvants, as a combination system, has increased potential to enhance vaccine immunogenicity, limiting side effects and optimising dose requirements, with cost-effective and simple formulation advantages.

Following the increased use of subunit and synthetic vaccines, the demand of higher property adjuvants has increased. The breakthrough of adjuvants from different strategies offers strong support for the adjuvant evolvement, and progress within these fields will gradually allow for a more comprehensive understanding of adjuvants and, consequently, safer, more effective vaccines.

## Figures and Tables

**Table 1:** Immunological adjuvants assessed as part of infectious disease vaccines.

Type	Adjuvant(s)
Mineral Compounds	Aluminum salt *
	Calcium salt
Microbial/Bacterial Products	Flagellin
	Lipopolysaccharide
	Cholera toxin (CT)
	Bacillus Calmette-Guérin (BCG)
Emulsions	CFA/IFA
	Montanides
	MF59 *
	GLA-SE
	TiterMax
	RIBI
Immunostimulatory complexes	Cytokines
	Chemokines
Particulate	Imidazoquinolines
	Virus-like particles/Virosomes
	Synthetic polynucleotides (e.g., CpG *)
	Liposomes
	Polysaccharides
	Polymeric nanoparticle adjuvants
	Glycosphingolipids (NK agonists)
Tesoactive	Saponin-based
Protease	Papain
Combination Adjuvant Systems (AS)	AS01 *, AS02, AS03 *, AS04 *

* Adjuvants used in licensed infectious disease vaccines.

**Table 2:** Vaccine adjuvants used in pre-clinical and clinical development against infectious disease pathogens.

Adjuvant Class	Immune Response(s)	Experimental Vaccine Details	Development Stage	Ref.
Mineral Adjuvants				
Aluminium salt (phosphate or hydroxide, Alum)	Enhanced cytokine, chemokine, antibody, and Th2 immune response	PiCoVacc (NCT04456595)	Phase 3	[38]
		BG505 SOSIP.664 gp140 (NTC04177355)	Phase 1	[39]
		Respiratory syncytial virus (RSV) (NCT01905215)	Phase 1	[40]
		RSV F vaccine (NCT01704365)	Phase 2	[41]
		RSV F vaccine (NCT02247726)	Phase 3	[42]
		HIV AIDSVAX B/E (NCT00006327)	Phase 3	[43]
		SCB-2019 (NCT04405908)	Phase 1	[44]
Calcium salt		Herpes simplex virus (HSV)-2 vaccine	Pre-clinical	[45,46]
		Human enterovirus-71 virus (HEV-71)		[47]
		Newcastle disease virus (RDVF) vaccine		[48]
		Foot and mouth disease virus (FMDV) DNA vaccine		[49]
Microbial/Bacterial Products—Flagellin				
Flagellin	Enhanced Th1 and Th2 immune responses (toll-like receptor [TLR] 5 agonist); pattern recognition receptor (PRR) activation; strong mucosal IgA/Th2/Th17 responses	VAX-102 (NCT00603811);	Phase 1	[25,50]
		VAX-125 (NCT00966238 and NCT00730457)	Phase 1	[51,52]
		VAX2012Q (NCT02015494)	Phase 1	[53]
		VAX2012Q (NCT02434276)	Phase 2	[25]
		Plague the flagellin-F1-V recombinant fusion protein (NCT01381744)	Phase 1	[54]
Microbial/Bacterial Products—Lipopolysaccharides				
Glucopyranosyl lipid adjuvant-aqueous formulation (GLA-AF)	Enhanced Th1 immune responses (TLR1/2 agonist)	Influenza H5-VLP vaccine (NCT01657929)	Phase 1	[55]
Microbial/Bacterial Products—Cholera toxin				
Cholera toxin (CT)	Enhanced Th1 and Th2 immune response	Recombinant *Naegleria fowleri* (rNfa1) protein-based vaccine	Pre-clinical	[56]
		Malaria (*Plasmodium vivax)* ookinete surface protein (OSP), Pvs25 (AdPvs25)		[57]
		HIV Envelope (Env)		[58]
Microbial/Bacterial Products—Bacillus Calmette-Guérin (BCG)				
Bacillus Calmette-Guérin (BCG)	Enhanced Th1 immune responses	Tuberculosis AERAS-404 and BCG revaccination with recombinant protein vaccines (H4:IC31 and H56:IC31) (NCT02378207; NCT02075203)	Phase 1/2	[59,60,61]
		COVID-19 vaccine (NCT04384549; NCT04328441; NCT04327206)	Phase 3	[62,63]
Emulsion—Freund’s Complete/Incomplete Adjuvants				
Incomplete Freund’s adjuvant (IFA)	Enhanced Th1-or mixed Th1/Th17 and Th1/Th2 type immune responses	HIV-1 immunogen emulsified in IFA adjuvant, which was conducted on 25 participates (NCT00381875)	Phase 1	[64]
Emulsion—Montanide				
ISA-51	Enhanced antigen uptake by antigen-presenting cells and potent stimulator of adjuvant core response genes, and cytokine, chemokine and Th2 (antibody) responses	Mosquito borne disease anopheles gambiae saliva vaccine (NCT03055000)	Phase 1	[65]
Emulsion—MF59^®^				
Squalene in oil-in-water emulsion (Squalene + Tween 80 + Span 85)	Enhanced Th1-or mixed Th1/Th17 and Th1/Th2-type immune responses	Seasonal influenza aIIV3; Fluad^®^ (NCT04576702)	Phase 2	[66]
		SARS-CoV-2 Sclamp antigen combined with MF59^®^ (NCT04495933)	Phase 1	[67]
Emulsion—GLA-SE				
Glucopyranosyl lipid adjuvant (GLA) in combination with squalene (SE)	Strong Th1 immune response	*Schistosoma* Sm14 antigen combined with GLA-SE adjuvant (NCT01154049)	Phase 1	[68]
		Tuberculosis ID93 antigen combined with GLA-SE adjuvant (NCT01599897)		[69]
Emulsion—TiterMax				
TiterMax^®^	Enhanced Th2 immune response	*Schistosoma mansoni* venom allergen-like protein with TiterMax adjuvant	Pre-clinical	[12]
Emulsion—RIBI Adjuvant System				
RIBI adjuvant system (RAS)	Enhanced Th2 immune response	Recombinant influenza viral nucleoprotein combined with RAS^®^ system	Pre-clinical	[70]
		*Mycobacterium paratuberculosis* 85B antigen of MPT combined with RIBI adjuvant system		[71]
Immunostimulatory Complexes—Cytokines				
Granulocyte-macrophage colony-stimulating factor (GM-CSF)	Enhanced Th1/Th2/CD8^+^ T and mucosal IgA responses; immune responses, activation of dendritic cells; increased migration and antigen presentation to CD4^+^ T cells; cross-priming of CD8^+^ T cells; generation of Th1-biased CD4^+^ T cells	BRII-179 combined with IFN-α (ACTRN12619001210167).	Phase 1	[72]
		HIV Vacc-C5, containing residues 489–511 from the HIV-1 virus C5 domain, with GM-CSF adjuvant (NCT01627678)	Phase 1/2	[73]
		Dendritic cells loaded with S-protein from SARS-CoV-2 alongside GM-CSF (NCT04386252)	Phase 1	[74]
Type 1 interferon (IFN)		BRII-179 combined with IFN-α (NCT04749368)	Phase 2	[72]
Interleukins (IL-1, IL-15, IL-2, and Il-18)		Influenza A viruses (H1N1 and H3N2) adjuvanted with INF-α (NCT00436046).	Phase 1	[75]
Immunostimulatory Complexes—Chemokines				
Chemokine CCL3	Secretion of mucosal IgA and CTL	HIV-1 Gag antigen with murine chemokine CCL3	Pre-clinical	[76]
Particulate—Imidazoquinolines				
Resiquimod (R-848)	Enhanced Th1 and Th2 immune responses (TLR7/8 agonist) and secretion of Type 1 IFN, pro-inflammatory cytokines, antibodies, and CD8^+^ T cells	Influenza IPR8-R848	Pre-clinical	[77]
Imidazoquinolines		HSV RSV vaccine candidate		[78]
Particulate—Virus-like Particles/Virosomes				
Virus-like particles (VLPs)	Enhanced Th1, Th2, and CD8^+^ T cell immune response	Nine-valent human papilloma virus vaccine (V503-020) (NCT02114385)	Phase 3	[79]
		Chikungunya virus VRC-CHKVLP059-00-VP (NCT02562482)	Phase 2	[80]
		Recombinant hemagglutinin trivalent nanoparticle vaccine (tNIV) produced in Sf9 insect cell-recombinant baculovirus platform containing wide-type virus sequences composed of conserved H3N2 epitope, as an influenza vaccine candidate (NCT03293498).	Phase 1/2	[81]
Virosomes	Enhanced Th1, Th2, and CD8^+^ T cell immune response	Combined HIV-1 virulence peptide antigens (gp41, p1) adjuvanted with virosomes (NCT04553016).	Phase 1	[82]
		Anti-malaria peptide vaccine derived from the circumsporozoite protein and apical antigen-1 (AMA-1) adjuvanted with virosomes (NCT00408668).	Phase 2	[83]
Double stranded (ds)-RNA (e.g., Poly I:C; PolyI:C_12_U (Ampligen^®^)	Type I IFN induction; pro-inflammatory cytokine/chemokine/antibody/CD4^+^/CD8^+^ T cell responses; mucosal adjuvant inducing Th1 and Th17 immune responses	H5N1 influenza antigen was a formalin-inactivated whole virus (NIBRG_14_) derived from a recombinant avian virus from H1N5 combined with Poly I: Poly C_12_ U (Ampligen^®^) adjuvant (NCT00711295)	Phase 3	[84]
		Combined plasma HIV-1 RNA with the TLR-3 agonist Poly-ICLC adjuvant as an HIV vaccine (NCT00207195).	Phase 1	[85]
RNA cyclic di-GMP oligodeoxynucleotide (CpG ODN) (e.g., unmethylated CpG DNA, CpG ODN; CpG 1018; CpG 7909; IC31)	PRR activation; potent inducer of ‘adjuvant core response genes’	Anthrax AVA vaccine (NCT01263691)	Phase 1	[86]
		Malaria vaccine (NCT00344539)	Phase 1	[87]
		Tuberculosis H4:IC31(ASERAS-404) (NCT02066428 and NCT02074956)	Phase 1/2	[88]
Particulate—Liposomes				
Cationic liposomes (e.g., CAF01)	Immunostimulatory depot effect; strong antigen-specific antibody and Th1/Th2 cell responses	COVID-19 mRNA-1273 vaccine—lipid nanoparticle-encapsulated with nucleoside-modified mRNA which encodes the SARS-CoV-2 spike (s) glycoprotein (NCT04470427)	Phase 3	[89]
Reformed liposomes		Tuberculosis hybrid 1(H1) fusion protein adjuvanted with CAF01 liposome (NCT00922363)	Phase 1	[90]
		*Chlamydia trachomatis* recombinant protein subunit CTH 522 adjuvanted with CAF01 liposome (NCT02787109)	Phase 1	[91]
Particulate—Polysaccharides				
Chitosan	Enhanced Th1 and Th2 immune response and mucosal immune response; site-directed delivery of antigens; source of DAMP (chitosan); mucosal (chitosan); induction of pro-inflammatory cytokines and secretory antibody responses; Th2 and mucosal IgA responses; PRR activation; upregulation of co-stimulatory molecules; activation of complement pathways.	Norwalk virus MPL/chitosan-VLP vaccine (NCT00973284)	Phase 2	[92]
AdvaxTM		Hepatitis B surface antigen (HBsAg) combined with Advax (ACTRN12607000598482)	Phase 1	[93]
		HIV-1 vaccine candidate plus Advax adjuvant (NCT00249106)	Phase 1	[94]
Particulate—Polymeric nanoparticles				
Polymeric nanoparticles e.g., PLA: poly (lactic acid); PLGA: poly (lactic-co-glycolic acid)	Enhanced Th1 and Th2 immune response; depot effect; mucoadhesive; strong antigen specific Th1/Th2, CD8^+^ T cell a and antibody responses	MPL/chitosan-VLP HIV vaccine (NCT00973284)	Phase 2	[92,95]
		HBV antigen (HBsAg) has been conducted into healthy adults (ACTRN12607000598482).	Phase 1	[93]
		HIV-1 vaccine candidate plus Advax adjuvant (NCT00249106)	Phase 1	[94]
Particulate—Glycosphingolipids				
Glycosphingolipids (α-GalCer)	Pattern recognition receptor activation; Th1/CTL and mucosal IgA responses	Combined HBV protein antigen with synthetic α-GalCer (KRN 7000), intravenously administrated into participants against HBV (NCT00363155).	Phase 2	[96]
Tensoactive—Saponin				
Matrix-MTM	Induction of cytokines and cellular influx; induction of Th1- or mixed Th1/Th17-, Th1/Th1-type as well as strong antibody (CD8^+^ T cell) responses	Matrix-M adjuvant combined with the full-length SARS-CoV-2 spike (S) glycoprotein (NCT04368988)	Phase 1/2	[97]
		COVID-19 NVX-CoV2373 (EudraCT number, 2020-004123-16)	Phase 3	[98]
Immune-stimulating complexes (ISCOMs)				
QS-21		H7N9 influenza (NCT01897701)	Phase 1	[99]
Protease—Papain-like Cysteine Proteases				
Papain-like cysteine proteases	Enhanced T helper (Th) 1 and Th17 immune responses	Schistosomiasis glyceraldehyde 3-phosphate dehydrogenase (SG3PDH), peroxiredoxin (TPX), and other larval excretory–secretory products (ESP) with Papain	Pre-clinical	[100]
Combination Adjuvant Systems				
AS01(MPL, QS-21 and liposome)	Transient induction of cytokines at the site of injection; increased influx of antigen-loaded monocytes in draining lymph nodes (dLNs)	RTS, S/AS01 malaria (NCT00866619)	Phase 3	[101]
AS02 (3-O-desacyl-4′-monophosphoryl lipid A [MPL] and QS-21)		Malaria FMP2.1/AS02A vaccine (NCT00460525)	Phase 2	[102]
AS03 (Squalene, polysorbate 80 and α-tocopherol)		SK SARS-CoV-2 recombinant protein nanoparticle vaccine (GBP 510) combined with AS03 (NCT04742738) and (NCT04750343)	Phase 1/2	[103,104]
		Influenza A (H1N1) pdm09 vaccines combined with AS03 (NCT00616928)	Phase 3	[105,106]
AS04 (MPL adsorbed onto aluminium hydroxide or aluminium phosphate)		HPV-16/-18 VLP adjuvanted with AS04 (NCT00128661)	Phase 3	[107]

## Data Availability

Not required.
